# Personality traits and bricolage as drivers of sustainable social responsibility in family SMEs: A COVID‐19 perspective

**DOI:** 10.1111/basr.12222

**Published:** 2021-03-01

**Authors:** Muhammad Anwar, Thomas Clauß

**Affiliations:** ^1^ Witten Institute for Family Business University of Witten/Herdecke Witten Germany

**Keywords:** big five personality traits, bricolage, COVID‐19, CSR, emerging markets, family firms, SMEs

## Abstract

Motivated by the social and environmental challenges resulting from the COVID‐19 pandemic, this research examines the influence of the “big five” personality traits; extroversion, agreeableness, openness, conscientiousness, and neuroticism on sustainable social responsibility with a mediating role of bricolage. We collected empirical evidence from 245 family‐owned SMEs. The results indicate that the personality traits do not directly influence sustainable social responsibility, although the traits (except extroversion) influence bricolage. Moreover, we found that open, conscious, and agreeable personalities indirectly contribute to sustainable social responsibility, with bricolage as a mediator. Our findings encourage enterprises to focus on those personality traits during crises (especially COVID‐19) that empower people to effectively manage existing resources (e.g., bricolage) and protect their stakeholders. Family‐owned SMEs need to assign resource utilization tasks to family members having personalities of openness, conscientiousness, and neuroticism because these kinds of people have high capacities for bricolage.

## 1 INTRODUCTION

One of the greatest challenges the modern world has faced is COVID‐19 pandemic. The disease is caused by syndrome coronavirus 2 (SARS‐CoV‐2), traced in a Chinese city Wuhan in December 2019. At the start of March, 2020, the World Health Organization acknowledged that the disease is spreading very quickly worldwide (Bretas & Alon, 2020). COVID‐19 has disrupted all organizational and individual activities across the globe. Businesses have seen their inward and outward internationalization transactions become interrupted and even clogged (Ratten, 2020). On the other hand, stakeholders are seeing institutional pressure directed at firms to take social and environmental action that benefits poor communities during the crisis. This second point has received tremendous attention from enterprises in Pakistan because of its collectivist culture, sense of social accountability, and the general need to support the poor. For instance, 43% of the population in Pakistan (57.2 million people) are laborers. Their income and earnings in some cases have literally dropped to zero during the COVID‐19 pandemic. In this situation, along with non‐profit organizations (NPOs), the industry is the second source that provides daily necessities such as food and clothing to the communities. Because of their number and presence in the country, SMEs are particularly seen as a foremost industry sector (Khan et al., 2019), with many of them starting social initiatives to benefit poor communities by providing food and clothing (Eggers, 2020; Lu et al., 2020). Some of these SMEs display a significant motivation toward social activities when compared to others, raising specific questions about them as a result.

SMEs are well‐known for their lacking resources which hinders their social practices. This is widely discussed in the literature (Bocquet et al., 2019; Ilyas et al., 2020). There is ample evidence indicating that SMEs exploit beneficial opportunities and try to manage their resources efficiently to participate in social activities (Khattak, 2020; Ilyas et al., 2020; Memon et al., 2020). Even though this is the case, the psychological factors and especially the personality traits of top managers and owners as they relate to social practices are often neglected. In a study, Berings and Adriaenssens (2012) considered the big five traits as control variables in the model of CSR activities only as dependent variables, revealing that personality plays a significant role in CSR activities. Otherwise, most of the research on psychological factors and environmental activities are limited to more narrow traits (e.g., social dominance orientation; emotional stability; cognitive, prosocial value orientation; system justification) (Arbuthnot, 1977; Basil & Weber, 2006; Hirsh & Dolderman, 2007). During COVID‐19, perhaps no study has yet focused on top managers’ personality factors in social activities. We argue that personality factors are important especially during COVID‐19 because all the people have not the same capabilities to bricolage (e.g., the capability of using existing resources effectively and efficiently), and no desire to participate in social activities. Additionally, what types of traits enable top managers to efficiently utilize resources (bricolage) remains under‐researched. Better insight into this topic would improve our understanding of standardized traits (e.g., the big five) that achieve sustainable social responsibility (SSR) and bricolage. SSR is an emerged term of CSR that covers various social activities concerning owners, employees, customers, suppliers, the community, and the environment. It is also not yet known what kinds of personality traits (extroversion, agreeableness, openness, conscientiousness, or neuroticism) lead SSR either directly or indirectly through a bricolage of resources. This study works to fill this gap, examining the influence of the big five personality traits on SSR with a mediating role of bricolage.

There are several motives/drivers behind using personality traits and bricolage as mediators toward SSR. First, other factors such as motivation (Grimstad et al., 2020), competition (Graafland & Noorderhaven, 2020), a global mindset (Paul et al., 2011), ability (García‐Sánchez et al., 2020), and the intellectual capital (Barrena‐Martinez et al., 2019) of top managers are discussed in the context of CSR, while the big five personality traits and how they impact SSR have been neglected, even though these traits play a significant role in organizational outcomes and success. Concerning COVID‐19, studies have never assessed the relationship between top managers’ psychological factors, bricolage, and social activities. Second, considering the COVID‐19 pandemic, it is difficult for organizations and especially SMEs to trade and collect resources (Eggers, 2020), compelling them to use their existing resources efficiently to obtain maximum benefit. In this case, bricolage plays an important role because it is concerned with effectively utilizing existing resources. This, however, has remained outside of the scope of research in the context of personality and SSR. Third, during the COVID‐19 pandemic, in particular, societies in Pakistan have increased pressure on business to profit socially instead of financially. Despite practicing at least some social or environmental activities, the industry still needs comprehensive SSR, protecting stakeholders such as employees, owners, communities, suppliers, and customers with respect to environmental activities.

Our research makes three major contributions to the literature. First, this research aims to contribute to studies on the growing COVID‐19 crisis that has challenged the business world and SMEs in particular. Second, pressure has increased on top management to emphasize and adopt SSR, and our research enables them to ascertain suitable personality traits for SSR and bricolage. Third, this research offers important insights for policymakers and practitioners who formulate strategies for the environment and society. The findings enable them to hire managers with the right personalities and high bricolage abilities who can effectively achieve SSR under the crisis.

## 2 THEORETICAL BACKGROUND

### 2.1 Personality traits

The big five personality traits propose five major dimensions of personality: extroversion, agreeableness, openness to experience, neuroticism, and conscientiousness (Costa & McCrae, 1992; Howard & Howard, 1995). Personality is defined as “the sum of ways in which an individual reacts to and interacts with others” (Robbins & Judge, 2016, p. 175). The big five personality model is accepted by most scholars and researchers (e.g., Ward et al., 2017), and the traits are used to measure individual personality. A high score of a specific trait displays strength within it (Schmitt, 2013).

Barrick and Mount, (1991) define these traits as under:

*Extraversion*: Includes sociability, talkativeness, assertiveness, optimism, and being cheerful and robust.

*Agreeableness*: Specifies personal warmth, altruism, sympathy, utility, and collaboration.

*Neuroticism*: Depicts moodiness, tenseness, anxiety, and timidity.

*Conscientiousness*: Is reflective of determination, strong will, fortitude, consistency, and promptness.

*Openness to experience*: Shows an active mind, aesthetic sympathy, favorite for variety, knowledgeable curiosity, and extensive cultural interest.

### 2.2 Bricolage

The French anthropologist Claude Lévi Strauss introduced the concept of bricolage in 1966/67, defining it as “using current resources to create new forms and order from tools and materials at hand” (Levi‐Strauss, 1966).

Lévi‐Strauss presented bricolage as an analogy demonstrating a specific mode in which human actors relate to their environment. He deemed resourcefulness as a function of knowledge about the environment, which is exhibited in a process of bricolage through which an individual combines and uses different resources he/she has at hand to solve problems and exploit new opportunities (Baker, 2007). Lévi‐Strauss differentiated bricolage from engineering, in which *Ingénieurs* first identify a challenge and then go out to find resources to solve it. *Bricoleurs* in contrast start with resources on hand to respond to their problem, applying them to fulfill their needs (Ferneley & Bell, 2006).

Bricolage refers to combining the resources on hand to solve problems and take advantage of opportunities (Baker & Nelson, 2005). This is contrasted with behaviors that involve resource acquisition to exploit new opportunities and address new situations (Duymedjian & Rüling, 2010). Witell et al. (2017) stated that bricolage is built on the following capabilities: (1) actively addressing resource scarcity; (2) efficiently using what is on hand; (3) extemporizing when recombining resources; and (4) relationships with external partners.

In the context of SMEs, scholars have defined entrepreneurial bricolage as “making do by creating new combinations of the resources at hand to new problems and opportunities.” Entrepreneurs make use of a variety of means and resources and engage in bricolage to gain maximum benefit and satisfy their customers. Considering the current COVID‐19 situation, we posit that bricolage has become even more vital for SMEs. For instance, SMEs already face resource constraints. The COVID‐19 crisis has attenuated trade, keeping it in some cases limited to using the resources that are on hand. As important as bricolage is for small enterprises due to their limited resources, it is also essential in emerging markets because enterprises there have scarce, poor resources—motivating businesses to recycle and mobilize their existing resources efficiently (Linna, 2013).

### 2.3 Sustainable social responsibility

The idea of CSR takes us back to Bowen (1953) who wrote the seminal book *Social Responsibilities of the Businessman*. Bowen defined social responsibility as “the obligations of businessmen to pursue those policies, to make those decisions, or to follow those lines of action which are desirable in terms of the objectives and values of our society.” It can perhaps be understood as a fact that, previously, social responsibility in business was voluntary; there essentially was no external pressure to adopt and practice social activities. However, as the business model changed (e.g., firms realized that social responsibility provides superior profit) (Carroll et al., 2012), and society became modern, customer pressure increased, legislation recognized different needs, and competition took place, social activities became a key source of competitive advantage. Since then there has been a shift in terminology from the social responsibility of business to CSR (Garriga & Melé, 2004). Organizations have gradually started understanding social activities as integral actions rather than mere volunteerism (Khan et al., 2020; Waller & Lanis, 2009). Businesses have realized that CSR is the key to competitive advantage (Porter & Kramer, 2006), reputation (Zhu et al., 2014), and financial performance (Choi et al., 2010). Consequently, the field has grown significantly, and today contains a wide selection of theories, terminologies, and approaches. For instance, stakeholder management, social policy, social issues management, society, and business, corporate accountability, SSR, and public policy and business are just some of the terms used to depict the phenomenon related to social responsibility in society (Garriga & Melé, 2004; Khan et al., 2020). Khan et al. (2020) claim that SSR, synonymous with CSR, is now an obligation of firms rather than something voluntary. Top managers should favor CSR activities to respond to crises (Sajko et al., 2020). They should manage activities properly to configure CSR activities (Endrikat et al., 2020; Lee et al., 2020).

According to stakeholder theory (Freeman & McVea, 2001), management should actively explore its relationships with all stakeholders (not only shareholders and owners) to develop business strategies and to satisfy their requests and needs (Shad et al., 2019). Under the best of circumstances, this can result in high performance (Martín‐de Castro et al., 2016; Singh et al., 2019) and a sustainable competitive position (Jones et al., 2018; Yadav et al., 2017). Parmar et al., (2010) have claimed the emerging theory of stakeholders to understand three interconnected business problems; (a) how value is created and traded, (b) connecting ethics and capitalism, and (c) helping managers think about management to solve the first two problems. Considering this theory in the current pandemic, we argue that top managers of SMEs search for possible solutions to create values while responding to the situations effectively. Concerning the Pakistan market, we demonstrate that social activities are considered essential for business firms due to the high pressure of the community. Hence, top managers of SMEs can create values through value activities in the market.

## 3 HYPOTHESES DEVELOPMENT

### 3.1 Personality traits and SSR

CSR activities are now considered an essential element of sustainable competitive advantage and firm performance (Jia, 2020; Saha et al., 2020). Although not all managers/owners and stakeholders are actively engaged in CSR activities, managers with certain abilities are. They are characterized by motivation (Basil, & Weber, 2006), the right political mindset (Jiang et al., 2018), competition and motives (Graafland & Noorderhaven, 2020), and certain demographic characteristics (Liu et al., 2018). Basil and Weber (2006) recommended that firms focus on managers with specific types of personality traits, and who are CSR‐oriented. Despite the ample evidence confirming the importance of CSR and environmental activities in business success and competitiveness, many SMEs are still reluctant to carry them out (Knight et al., 2019). Major reasons behind their lack of interest include limited resources, poor support (Khattak, 2020), and lack of stakeholders’ interest (Albats et al. 2019).

Stakeholder theory is widely known in the literature. It sheds light on firms handling their accountabilities in a socially responsible manner to consider and look after their stakeholders (McGuire et al., 2003). Shareholders and CEOs are the primary persons caring for their organizations. Their characteristics significantly influence the activities of financially driven organizations (Carmeli et al., 2020). In the case of SMEs, top managers and owners are considered as the top managers who have influenced organizations’ outcomes. For instance, top management characteristics, background, and psychological factors affect organizational activities, policies, and outcomes (Hambrick, 2007). The claim of the UET is supported empirically and theoretically in business and marketing literature. For instance, Pérez and del Bosque (2017) scrutinized the demographic characteristics of age, experience, gender, and education, finding that they have dissimilar relationships to CSR activities. In general, the big five traits influence the citizenship behaviors of employees in an organization (Organ, 1994).

We realized that very limited evidence exists regarding the relationship between CEOs’, executives’, and top managers’ traits and CSR activities. The limited literature that does exist claims that there is a significant relationship between personality traits and environmental behavior (Arbuthnot, 1977; Fraj & Martinez, 2006). However, in particular, it does not answer which personality traits influence SSR among family firms?”.

On the other hand, Myung et al. (2017) scrutinized the negative traits of CEOs: Machiavellianism, psychopathy, and narcissism, which are inversely related to CSR activities in organizations. Research has revealed fragmented results on the relationship between personality traits and environmental activities. Many specific facets of personality have been scrutinized over the years, identifying traits such as extroversion, agreeableness, and conscientiousness as favorable, while neuroticism is an example of a negative predictor of environmental initiatives (e.g., Borden & Francis, 1978; Milfont & Sibley, 2012). Generally, being extroverted, agreeable, conscientious, and open to experiences are characterized as positive drivers of environmental activities (Fraj & Martinez, 2006; Hirsh, 2010; Markowitz et al., 2012).

It is argued that the trait of extroversion is evidence of higher pro‐environmental activities (Borden & Francis, 1978). CSR activities are something done extra to achieve social aims and build communities (Lindgreen et al., 2009). This means that proactive behaviors are required for performing environmental deeds (Cheong et al., 2017). Fang et al. (2015) revealed in their meta‐analysis that extroverted individuals are more socially oriented and tend to build broader ties with people. Extroverted people are interested in social engagement and commitment (Malhotra et al., 2018), which is why it is argued that managers and owners with extroverted personalities are likely to engage in SSR.

Tran and Von Korflesch (2016) also claimed that highly extroverted, agreeable, open, and conscientious people have high socio‐entrepreneurial intentions, while neurotic individuals have a low level of them. Agreeable persons display strong environmental behavior and are engaged in environmental activities (Hirsh & Dolderman, 2007). High levels of openness make people tend to be engaged in new actions and likely to practice new activities, while a lower level of openness keeps individuals from social work, with them tending to prefer conventional and traditional practices (Howard & Howard, 1995).

It is argued that relatively stable individual difference factors may play a significant role in shaping environmentally relevant tendencies and factors. For instance, some personality constructs motivate individuals toward pro‐environmental action (Markowitz et al., 2012). People that are extroverted, neurotic, and open to experienced have more social interests, making them more likely to use social media such as Facebook (Hughes et al., 2012; Wang et al., 2012). Tang et al. (2016) on the other hand examined how personality traits such as agreeableness, conscientiousness, and neuroticism are negatively associated with addiction to Facebook. Wee et al. (2017) revealed that high levels of neuroticism lead to a low level of social networking among individuals. Similarly, Mandal et al. (2017) tested the relationship between the personality traits of owners of small enterprises and social media usage, describing that the relationship here varies from trait to trait. To summarize, after reading the literature, we realized that the relationships between social personality factors and social activities display fragmented characteristics as they relate to green decision making and environmental involvement (Busic‐Sontic et al., 2017; Tang et al., 2016). These results make it difficult to state which type of personality traits lead to SSR. However, taking just one example of extroverts, that is, people who are interested in building and engaging in social networking, have a high interest in social interaction, are talkative, and like to engage in social tasks (Costa & McCrae, 1992), we see that these kinds of individuals are willing to do new things like adapt to new situations, and have a high capacity for understanding them (Costa & McCrae, 1992). These kinds of people are furthermore concerned about the welfare of others and enjoy performing selfless service in their careers (Costa & McCrae, 1992). Openness people are more politically oriented and have high environmental behaviors (Klein et al., 2019). Moreover, a few studies have reported openness to experience as the most predictor of pro‐environmental attitudes and activities (Brick & Lewis, 2016; Markowitz et al., 2012; Soliño & Farizo, 2014). People score high in agreeableness cooperate with others and have a high desire for social compatibility (McCrae & Costa, 1997). Agreeable people show empathy which positively influences pro‐environmental behaviors (Hirsh & Dolderman, 2007). Individuals with neurotic traits on the other hand have low confidence, like to live within a small circle of people, and tend to be lonely instead of working as leaders of social change (Costa & McCrae, 1992). People with neuroticism traits do not tend to participate in environmental initiatives (Milfont & Sibley, 2012). Conscientious individuals are competent and dutiful, like to achieve their goals and tasks, and are involved in organizing things (Costa & McCrae, 1992). Conscientious people are interested in social and environmental initiatives (Fraj & Martinez, 2006; Tran & Von Korflesch, 2016). We, therefore, hypothesize:

H 1 Being extroverted is positively related to SSR.

H 2 Being open is positively related to SSR.

H 3 Being agreeable is positively related to SSR.

H 4 Being neurotic is negatively related to SSR.

H 5 Being conscientious is positively related to SSR.

### 3.2 Personality traits and bricolage

Personality is understood as peoples’ attitudes, behaviors, stable motives, experiences, and directed actions (Meyer et al., 2014). Personality is also reflected upon to shape the behavior and attitudes of individuals in any society (Lim & Melissa Ng Abdullah, 2012). Every individual has different traits. For instance, in a workplace, some individuals prefer to work in a group, more sociably, participate in every activity, and manage things, while others like to perform their tasks alone, do not like to sit in groups, and are less likely to participate in social activities.

Extroverted individuals are highly active, talkative, social, and assertive (Barrick & Mount, 1991). They are leaders and aim to energetically apply resources and capabilities to complete tasks (Costa & McCrae, 1992). Neurotic people are introverted, afraid to bring change and do not try new things, and perform tasks in an organized manner rather than stray from standard procedures. They display a low level of innovative behavior (Ali, 2019). Farrukh et al. (2017) revealed that neuroticism is negatively related to affective commitment.

Individuals with conscientious personalities are goal‐oriented and think before acting, planning, and organizing. They are more dependable, organized workers who effectively achieve tasks (Barrick & Mount, 1991). They immerse themselves in their work and tasks, requiring minimal requirements to achieve them (Organ & Ryan, 1995).

Individuals with agreeable personalities are helpful, forgiving, cooperative, and courteous (Barrick & Mount, 1991). Agreeable people enjoy engaging with other pleasant, satisfying people to create relationships with high levels of emotional attachment, generating extensive benefits (Matzler et al., 2011). Farrukh et al. (2017) state how agreeable people are actively engaged in affective commitment and tend to manage things in a better way to fulfill it (Ali, 2019).

Esmaeelinezhad and Afrazeh (2018) examine how agreeable, conscientious, extroverted, and open personalities achieve significantly managed knowledge in organizations, while neuroticism has a negative influence on knowledge acquisition and sharing behaviors. Extroverts are confident in their business activities. Confident managers can manage and increase earnings and decrease future losses (Berry‐Stölzle et al., 2018). Wang and Chen (2020) also determined that some traits (extroversion and agreeableness) are more important for cost efficiency and profitability when compared to neurotic managers. Openness to experiencing individuals involves a high level of risk‐taking and frequent involvement in uncertain activities. These personalities have a high ability to manage assets (e.g., financial resources) and entrepreneurship activities (Brown & Taylor, 2014; Obschonka et al., 2013). We therefore hypothesize:

H 6 Being extroverted is positively related to bricolage.

H 7 Being open is positively related to bricolage.

H 8 Being agreeable is positively related to bricolage.

H 9 Being neurotic is negatively related to bricolage.

H 10 Being conscientious is positively related to bricolage.

### 3.3 Bricolage and SSR

As an emerging entrepreneurship theory in the literature, bricolage has been considered an effective tool for organizations to overcome resource constraints (Baker & Nelson, 2005). Bricolage has strong implications for enterprises (Senyard et al., 2014). Desa (2012) states that entrepreneurs who use bricolage perform best practices of material inputs and resources. Resource constraints are the major problem hampering SMEs from environmental and social practices (Ilyas et al., 2020), which is why they need to manage resources effectively to perform them (Granados et al., 2017). In this sense, bricolage is the best strategy for SMEs to efficiently use resources to exploit new opportunities and gain performance (An et al., 2018). Steffens and Senyard (2009) describe how entrepreneurial bricolage provides better resource advantages, resulting in higher levels of competitive advantage.

Enterprises need to protect their resources to achieve long‐term competitiveness. Enterprises also need to incorporate social responsibility activities into their business operations (Khan et al., 2020; Zain et al., 2006) and be able to develop solutions by accumulating resources. Entrepreneurial bricolage can be a strong advantage in doing this (Hooi et al., 2016). Mzembe et al. (2019) revealed that institutional bricolage is a significant driver of social value creation in developing economies. Moreover, Kwong et al. (2017) claimed that bricolage enables social enterprises when they articulate their resources, thereby resulting in high performance. We claim that bricolage influences SSR because it assists SMEs in using their scarce, limited, and rare resources efficiently, enabling them to achieve SSR. For example, bricolage helps SMEs improvise, cope with difficulties, and accelerate new information system development (Ferneley & Bell, 2006). SMEs aim to meet the social needs of people with limited resources. In this context, bricolage facilitates them in overcoming challenges, enabling them to fulfill social needs (Kannampuzha & Suoranta, 2016). Therefore:

H 11 Bricolage is positively associated with SSR.

### 3.4 Bricolage as a mediator

Considering the model, we use bricolage as a mediator between personality traits and SSR in SMEs. It here should be noted that not all managers can effectively perform bricolage activities. Instead, certain characteristics can lead to bricolage and might result in social and environmental activities. For instance, Gupta et al. (2019) claimed that specific personality traits encourage managers toward CSR for several reasons. Moreover, Hossain and Asheq (2020) revealed that proactive personality traits significantly motivate students to become social entrepreneurs, and prior experience and management strengthens this pathway. We as a result perceive that bricolage (being a parameter of knowledge, experience, and management of resources) plays a key affecting the role between personality traits and social activities. Similarly, An et al. (2018) examined how young and mature firms have different influences on bricolage that result in high levels of innovation. Similarly, Yan et al. (2020) also revealed that top management boundary‐spanning behavior significantly influences bricolage to build an effective business model. This notion is also supported by Su et al. (2020) who claimed that top management team heterogeneity effects entrepreneurial bricolage in newly born ventures. Considering the evidence, we argue that top managers’ psychological traits can influence bricolage activities in family SMEs.

Enterprises often face resource constraints when they work to build their business, improve performance, and operate among transitional circumstances. In this sense, top managers need to emphasize bricolage strategy to avoid resource constraints and achieve objectives (Yan et al., 2020). Most entrepreneurs start with limited fundamental resources (Baker & Nelson, 2005), gradually mature and grow, then accumulate resources from external sources (Peng & Luo, 2000). In the development and maturity stage, enterprises are more actively engaged in social activities and environmental practices compared to new and young firms (Withisuphakorn & Jiraporn, 2016). It is also well known in the literature that many SMEs do not voluntarily engage in CSR activities because of resource constraints, poor support, and lacking capabilities (El Baz et al., 2016; Ilyas et al., 2020). It alternatively can be very beneficial to top SME managers to first efficiently manage resources and recognize opportunities before practicing CSR (Memon et al., 2020). The top management has a significant influence on firms’ policies, resources, and strategies that lead to desired performance and outcomes (Carpenter et al., 2004).

Studies have, however, generated mixed results in terms of personality traits and social initiatives. For instance, Nga and Shamuganathan (2010) displayed that agreeableness significantly influences socio‐entrepreneurship, while openness shows only a weak association with it. İrengün and Arıkboğa (2015) also described that agreeableness affects socio‐entrepreneurship, neuroticism influences resource use, openness to experience impacts social vision, and extroversion affects financial returns and resource use. Tran and Von Korflesch (2016) claimed that individuals with high extroversion, openness, conscientiousness, and agreeableness scores have high socio‐entrepreneurial intentions, but a low level of neuroticism. On the other hand, İrengün and Arıkboğa (2015) revealed that people with highly agreeable traits do not aim to participate in socio‐entrepreneurship activities that require struggle and, as a result, would likely take more time to even begin them. Moreover, Liang et al. (2015) also inspected the idea of how openness to experience new things harms socio‐entrepreneurial services. In emerging economies, bricolage is the key to managing and using existing resources, thereby enabling top managers to reach high levels of sustainable social performance (Sivathanu & Pillai, 2019). We, therefore, posit that some managers with specific traits might be more capable of managing resources to practice environmental and social activities when compared to the managers displaying a lack of motivation and desire for social activities.

H 12 Bricolage mediates the relationship between extroversion and SSR.

H 13 Bricolage mediates the relationship between openness to experience and SSR.

H 14 Bricolage mediates the relationship between agreeableness and SSR.

H 15 Bricolage mediates the relationship between neuroticism and SSR.

H 16 Bricolage mediates the relationship between conscientiousness and SSR.

**FIGURE 1:**
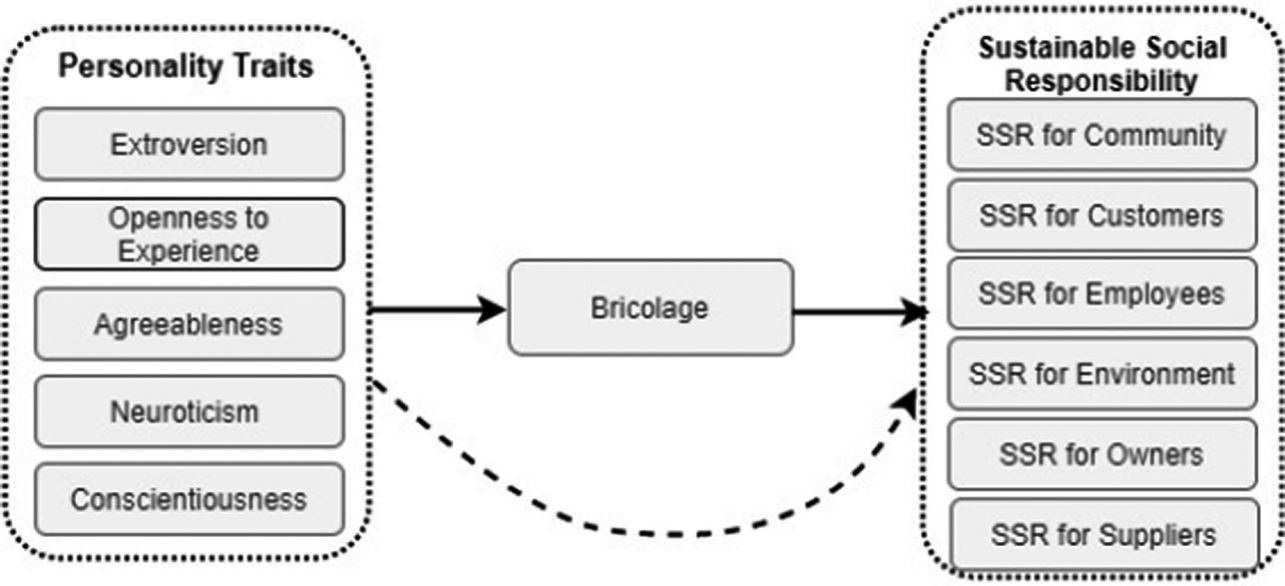
Conceptualized research model

Figure 1 illustrates conceptualized research model where the influence of personality traits on SSR with a mediating role of bricolage is shown.

## 4 METHODOLOGY

### 4.1 Sample and data

This study's empirical analysis is based on data from family firms working in the emerging market of Pakistan. The sample was composed of enterprises operating in four major cities there: Peshawar, a historical city of trade; Rawalpindi, a large industrial city; Islamabad, the capital of Pakistan; and Lahore, the trade center. These cities were selected because their head offices and centralized decision‐making processes of the family firms we examined are located within them. They are furthermore well‐known when it comes to business.

Given the aim of the study, only‐family‐based SMEs were targeted for our data collection. The literature has not yet determined a universal definition of SMEs (Memon et al., 2020), and every country defines it in its way (Beck et al., 2005). Generally, SMEs are defined based on annual sales, turnover, and number of employees (Anwar, 2018); due to the limitations of financial reporting and data, it is difficult to define SMEs based solely on financial figures. So some studies differentiate SMEs from other firms based on the number of employees (Anwar, 2018; Khan et al., 2019; Ilyas et al., 2020). In Pakistan, firms are considered SMEs when more than 20 and less than 250 employees work there (Anwar, 2018). With this definition in mind, we focused on those ventures where less than 250 employees are employed. It is estimated that two‐thirds of enterprises in Pakistan are family‐owned. Similarly, family firms have not a single definition. We considered those firms as family firms whose decision making process is influenced by family members or where family members were working as main actors (Arregle et al., 2007), and having employees less than 250 (e.g., family SMEs).

In Pakistan, SMEs are registered with the Chamber of Commerce and Small and Medium Enterprises Development Authority (SMEDA). We placed requests with the Sarhad Chamber of Commerce (3200 firms), Rawalpindi Chamber of Commerce (7900 firms), Islamabad Chamber of Commerce (6100 firms), and Lahore Chamber of Commerce and Industry (5900 firms) for business register lists, receiving the lists of registered firms. This contained information on each enterprise such as the name of the owner/manager, cell phone number, email, address, and kind of business. Each list had both family and non‐family firms.

We used a structured questionnaire with two sections for data collection. In the first section, we asked questions about the main variables such as personality traits, bricolage, and SSR. In the second section, we asked about demographic information such as the educational background, experience, and age of the owners; as well as the nature of their industry, its size, and the age of its enterprises. A cover letter was provided explaining how data secrecy would be ensured. It was also mentioned in the cover letter that the survey was to be completed by top managers/owners or assistant managers. Additionally, it was mentioned in the survey that only family‐owned firms or firms where the decision‐making process was done by family members would be allowed to participate in the survey. Because all the enterprises at the time of the research had closed their offices during the COVID‐19 lockdown, we used an online survey to collect data for the research. The online survey was thus the only source of data for the analysis. The good news from this is that during the lockdown, most of the enterprises did prefer contact via email and telecommunications. We enlisted enumerators who were paid for their services per questionnaire. They were guided in terms of the unit of analysis, target respondents, and procedure before data collection. We requested they distribute a maximum number of questionnaires in each city to obtain as many responses as possible. Enumerator A, who was assigned the data collection from Peshawar, sent 870 emails randomly (one to each firm) of which he received only 93 back, with 87 useful responses, comprising a response rate of 10%. Enumerator B was assigned the collection of evidence from Lahore and sent 1350 emails. She received only 73 useful responses with a response rate of 5.41%. Enumerator C collected data from the family firms operating in Islamabad and contacted 1200 firms via email and phone. He received only 85 useable responses with a response rate of 7.08%. This resulted in a total of 245 effective responses that were used for the research analysis. Table 1 illustrates the family SMEs who completed the survey.

**TABLE 1:** Profile of the family SMEs

Particulars	Frequency	Percent
*Industry*		
1. Manufacturing	93	38.0
2. Trading	64	26.1
3. Services	88	35.9
*Size of the family firms*		
1. 20–50 employees	29	11.8
2. 51–100 employees	38	15.5
3. 101–150 employees	62	25.3
4. 151–200 employees	71	29.0
5. 201–250 employees	45	18.4
*Age of the family firms*		
1. 10 years and less	52	21.2
2. 11–20 years	68	27.8
3. 21 and 30	71	29.0
4. 31 and above	54	22.0
*Qualification of top management/owners*		
1. Bachelors or less	82	33.5
2. Masters	89	36.3
3. MS/MPhil	58	23.7
4. PhD	16	6.5
Total	245	100.0

### 4.2 Measures

Our study used adapted measures with a 5‐point Likert scale displaying: strongly disagree (1) to strongly agree (5).

#### 4.2.1 Personality traits

While several personality traits have been introduced into the literature over the years (Myers et al., 2010), scholars and academia gradually merged to consolidate the concepts into the “big five” traits (Brown & Taylor, 2014; Goldberg, 1993; Schmitt et al., 2007). In the present study, we used these five traits of extroversion, agreeableness, openness, conscientiousness, and neuroticism that are taken from the study by Anwar et al. (2018). Sample items here include extroversion (“I am a cheerful, high‐spirited person”), agreeableness (“I generally try to be thoughtful and considerate”), openness (“I often try new and foreign foods”), conscientiousness “I keep my belongings neat and clean”), and neuroticism (“I often feel tense and jittery”).

#### 4.2.2 Bricolage

This is not a single measure of bricolage, even though there is strong consistency between the ways it is operationalized (Salunke et al., 2013). Instead of using only three items (Salunke et al., 2013), we used eight to measure bricolage; these are taken from a recent study by Zhao et al. (2020) who comprehensively discussed it. A sample item here is “We are confident in our ability to find workable solutions to new challenges by using our existing knowledge.”

#### 4.2.3 Sustainable social responsibility

Several measures are used in the literature for SSR. These include CSR, social activities, environmental activities, and sustainable practices (Jamali, 2008; Javed et al., 2017; Khan et al., 2020). In the present study, we aimed to use comprehensive measures of SSR that include building favorable ties with all stakeholders such as employees, owners, suppliers, customers, and the community to protect the environment. We used 62 items, of which 10 were for each SSR, including 12 for SSR toward customers; these are taken from a recent study conducted by Khan et al., (2020). Sample items for SSR include employees (“A healthy and safe work environment”), the environment (“Waste management”), the community (“Community volunteer programs”), customers (“Full product disclosure”), suppliers (“Utilize local suppliers”), and owners (“Encouraging staff ownership of shares”).

### 4.3 Control variables

We controlled the size and age of the family firms and the qualifications of their top managers/owners in our analysis to rule out alternative explanations. The reason for this was that larger and more mature firms have more resources for social activities when compared to small and newly founded enterprises. Moreover, it is argued that managers with high levels of education might strive to achieve more social practices as a result of their awareness of and knowledge about social efforts and their benefits. Consequently, studies in the same vein have also suggested these factors as controls when examining SMEs’ performance, outputs, and consequences (Anwar, 2018; Khan et al., 2020; Khattak, 2020). In our results, we found mixed results for these controls (see the findings of the structural model).

## 5 DATA ANALYSIS AND RESULTS

We executed our analysis in SmartPLS, with several screening tests including descriptive statistics, multicollinearity, common method bias, and non‐response bias; these were done using SPSS. SmartPLS is better than other software because it provides several benefits; separates measurement errors, works on small sample size and abnormal data, manages a complex model easily and better in the case of mediation models (Hair et al., 2017).

### 5.1 Descriptive statistics

We executed descriptive statistics on the constructs as shown in Table 2. Here it is seen that consistent with the suggestions by George (2011), our data and all factors are normally distributed concerning the acceptable values (±2) of skewness and kurtosis.

**TABLE 2:** Descriptive analysis

Constructs	Median	Kurtosis	Skewness	Multicollinearity	
				Bricolage	SSR
Extroversion	−0.037	0.495	−0.629	1.209	1.262
Agreeable	0.061	1.518	−0.606	1.320	1.364
Openness	0.059	0.913	−0.627	1.705	2.979
Conscientious	0.219	1.205	−0.689	1.284	1.626
Neuroticism	0.000	0.308	0.105	1.442	1.539
Bricolage	0.206	1.166	−0.499	–	2.077
SSR	0.170	1.585	−0.384	–	–
SSR Community	0.130	1.079	−0.202	–	–
SSR Customers	0.126	0.760	−0.200	–	–
SSR Employees	0.040	0.747	0.211	–	–
SSR Environment	0.109	0.923	−0.131	–	–
SSR Owner	0.135	1.177	−0.290	–	–
SSR Suppliers	0.100	0.663	−0.145	–	–

### 5.2 Multicollinearity

The problem of overlapping between independent variables that are loaded on a particular variable (dependent) is seen. The results are influenced as a result (Mansfield & Helms, 1982). We checked the influence of personality traits and bricolage on SSR in SPSS, applying the collinearity test to assess multicollinearity. This resulted in a variance inflation factor (VIF) below 3 and a tolerance above 0.10 for all of the constructs, confirming the absence of multicollinearity in the data (Schroeder et al., 1990).

### 5.3 Common method variance

This problem is very common in the cross‐sectional data where a researcher collects data through a survey while targeting the same respondents (MacKenzie & Podsakoff, 2012). Generally, a survey related to the success and failure of organizational operations is influenced by social desirability bias. To check for the CMV problem, Harman's single factor test was applied in SPSS where all the items of personality traits, bricolage, and SSR were included. The results displayed the first factor with a variance of 18.75%. This is below 50%, thereby confirming the absence of CMV in the data.

### 5.4 Measurement model

**FIGURE 2:**
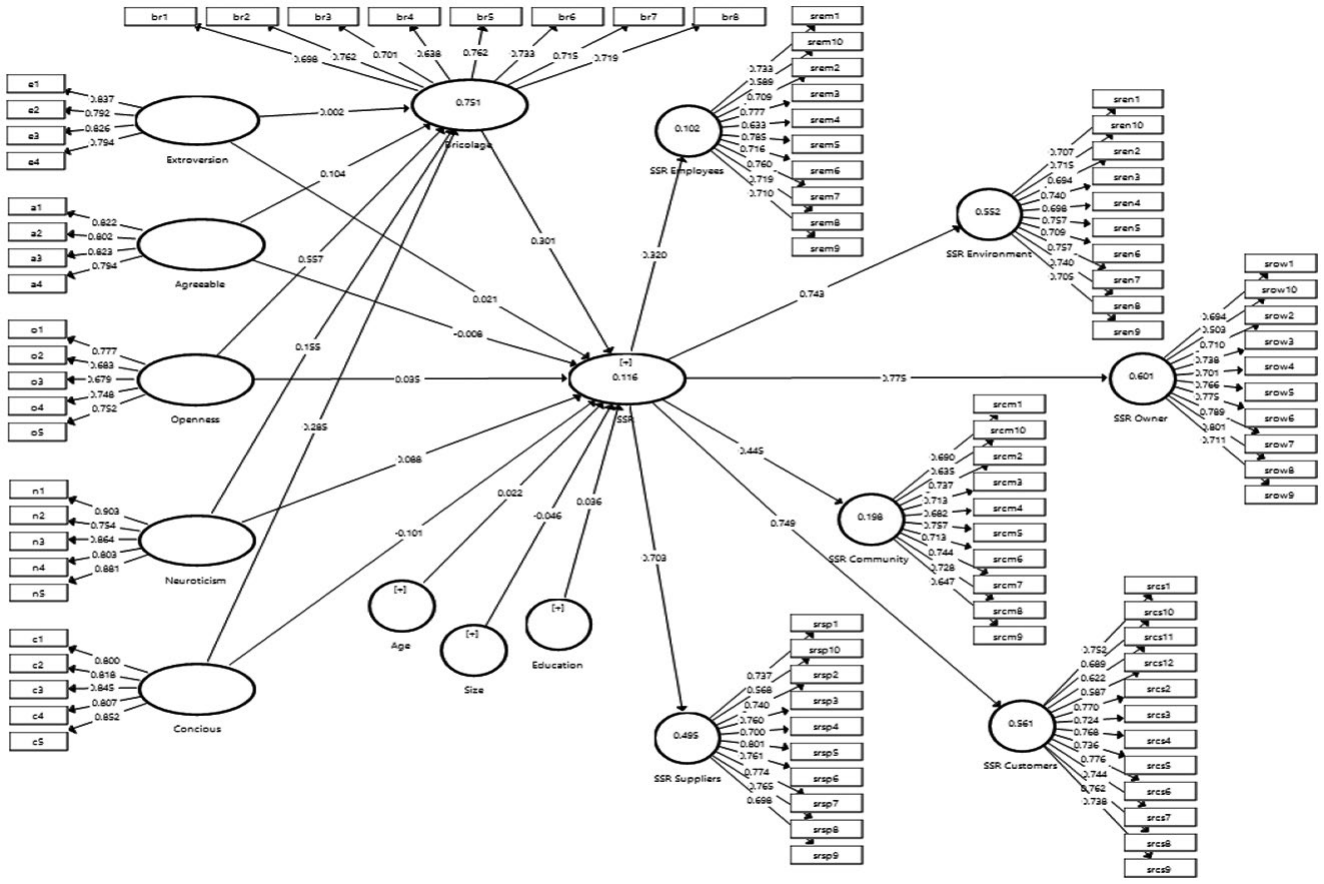
Measurement model

In this test (Figure 2), we ensured the factor loadings, validity, and reliability of the items and constructs. First, we checked the fitness of the model, confirming that SRMR below 0.10 and NFI above 0.90 is adequate (Browne and Cudeck (1992) recommended this value to be below 3 for a good model fit). All the items have satisfactory factor loading (above 0.70), and no major cross‐loading was reported between the items (see Table 3). The results of validity and reliability are shown in Table 4.

**TABLE 3:** Cross‐loadings

Cross‐loading	1	2	3	4	5	6	7	8	9	10	11	12
a1	**0.822**	0.322	0.166	0.264	0.191	0.261	0.122	0.063	0.02	0.076	0.045	−0.032
a2	**0.802**	0.356	0.222	0.366	0.276	0.346	0.165	0.071	−0.05	0.135	0.138	−0.016
a3	**0.823**	0.379	0.204	0.261	0.262	0.307	0.142	0.09	0.046	0.06	0.084	0.047
a4	**0.794**	0.328	0.151	0.302	0.167	0.333	0.209	0.1	0.029	0.133	0.127	−0.033
br1	0.24	**0.698**	0.364	0.229	0.409	0.486	0.206	0.149	0.048	0.231	0.193	0.142
br2	0.287	**0.762**	0.466	0.145	0.441	0.661	0.221	0.05	0.045	0.27	0.198	0.054
br3	0.291	**0.701**	0.406	0.208	0.343	0.543	0.108	0.136	0.068	0.219	0.152	0.077
br4	0.305	**0.638**	0.326	0.247	0.432	0.569	0.212	0.112	0.143	0.268	0.213	0.118
br5	0.345	**0.762**	0.495	0.195	0.423	0.636	0.168	0.113	0.01	0.297	0.238	0.096
br6	0.345	**0.733**	0.405	0.122	0.436	0.613	0.196	0.076	0.033	0.184	0.162	0.058
br7	0.315	**0.715**	0.431	0.138	0.365	0.555	0.205	0.113	0.054	0.274	0.183	0.123
br8	0.323	**0.719**	0.5	0.17	0.423	0.526	0.228	0.099	−0.024	0.169	0.165	0.035
c1	0.155	0.46	**0.80**	0.267	0.311	0.334	0.086	−0.003	−0.013	0.13	0.13	0.002
c2	0.186	0.506	**0.818**	0.213	0.208	0.369	0.127	0.054	−0.056	0.149	0.149	0.054
c3	0.208	0.502	**0.845**	0.179	0.252	0.365	0.062	0.055	−0.036	0.118	0.08	0.016
c4	0.177	0.474	**0.807**	0.209	0.191	0.355	0.116	0.002	−0.078	0.103	0.134	0.047
c5	0.223	0.506	**0.852**	0.226	0.272	0.339	0.113	0.037	−0.14	0.133	0.09	0.001
e1	0.36	0.201	0.265	**0.837**	0.121	0.135	0.087	0.039	−0.083	0.119	0.057	0.037
e2	0.264	0.199	0.159	**0.792**	0.22	0.173	0.084	0.059	−0.085	0.037	0.025	−0.073
e3	0.302	0.201	0.241	**0.826**	0.171	0.159	0.136	0.079	−0.042	0.066	0.116	0.057
e4	0.271	0.216	0.189	**0.794**	0.098	0.163	0.174	0.106	−0.07	0.092	0.005	−0.07
n1	0.181	0.5	0.273	0.178	**0.903**	0.484	0.121	0.126	0.032	0.236	0.194	0.088
n2	0.311	0.483	0.25	0.157	**0.754**	0.464	0.182	0.073	0.086	0.244	0.101	0.061
n3	0.192	0.466	0.23	0.17	**0.864**	0.452	0.093	0.119	0.061	0.245	0.18	0.077
n4	0.297	0.48	0.228	0.126	**0.803**	0.425	0.189	0.104	0.067	0.26	0.127	0.073
n5	0.198	0.474	0.272	0.145	**0.881**	0.454	0.104	0.128	0.011	0.246	0.175	0.102
o1	0.282	0.634	0.347	0.114	0.44	**0.777**	0.187	0.077	0.111	0.248	0.177	0.03
o2	0.221	0.572	0.296	0.17	0.292	**0.683**	0.13	0.112	0.124	0.196	0.107	0.087
o3	0.284	0.52	0.247	0.192	0.425	**0.679**	0.149	0.134	0.185	0.224	0.175	0.097
o4	0.292	0.588	0.353	0.161	0.382	**0.748**	0.141	0.109	0.062	0.21	0.199	0.074
o5	0.329	0.608	0.309	0.076	0.433	**0.752**	0.149	0.081	0.096	0.183	0.135	0.061
srcm1	0.162	0.215	0.128	0.141	0.118	0.141	**0.69**	0.063	−0.002	0.231	0.207	0.109
srcm10	0.208	0.186	0.093	0.128	0.154	0.14	**0.635**	0.053	0.001	0.118	0.091	0.052
srcm2	0.148	0.159	0.038	0.006	0.099	0.123	**0.737**	0.125	−0.023	0.241	0.243	0.133
srcm3	0.098	0.136	0.131	0.12	0.096	0.084	**0.713**	0.107	−0.047	0.215	0.156	0.168
srcm4	0.162	0.135	0.031	0.092	0.03	0.121	**0.682**	0.122	0.064	0.219	0.236	0.152
srcm5	0.092	0.195	0.122	0.14	0.142	0.114	**0.757**	0.114	0.017	0.254	0.22	0.204
srcm6	0.155	0.222	0.057	0.146	0.141	0.202	**0.713**	0.115	−0.059	0.262	0.205	0.119
srcm7	0.09	0.262	0.143	0.106	0.158	0.187	**0.744**	0.095	0.027	0.236	0.165	0.171
srcm8	0.183	0.222	0.047	0.145	0.139	0.175	**0.728**	0.073	−0.009	0.223	0.216	0.055
srcm9	0.133	0.181	0.081	0.04	0.094	0.207	**0.647**	0.149	−0.015	0.11	0.089	0.078
srcs1	0.025	0.038	−0.012	0.079	0.018	0.082	0.05	**0.752**	0.077	0.29	0.406	0.206
srcs10	0.103	0.129	0.019	0.029	0.058	0.131	0.079	**0.689**	0.141	0.347	0.476	0.441
srcs11	0.031	0.19	0.087	0.049	0.172	0.136	0.181	**0.622**	0.111	0.289	0.412	0.279
srcs12	0.116	0.218	0.111	0.084	0.1	0.172	0.245	**0.587**	0.078	0.303	0.459	0.41
srcs2	0.05	0.057	0.016	0.045	0.104	0.112	0.077	**0.77**	0.09	0.321	0.406	0.267
srcs3	0.049	0.047	0.019	0.122	0.026	0.017	0.066	**0.724**	0.136	0.209	0.392	0.223
srcs4	0.079	0.034	−0.015	0.036	0.063	0.05	0.113	**0.768**	0.099	0.246	0.418	0.303
srcs5	0.049	0.035	−0.037	0.028	0.087	0.016	0.033	**0.736**	0.176	0.189	0.38	0.181
srcs6	0.084	0.076	−0.005	0.071	0.088	0.09	0.116	**0.776**	0.076	0.2	0.388	0.278
srcs7	0.091	0.139	0.02	0.094	0.216	0.133	0.088	**0.744**	0.055	0.228	0.43	0.267
srcs8	0.109	0.124	0.045	0.079	0.081	0.137	0.081	**0.762**	0.01	0.2	0.379	0.257
srcs9	0.073	0.158	0.051	0.055	0.117	0.106	0.109	**0.738**	0.042	0.213	0.427	0.263
srem1	−0.009	0.03	−0.087	−0.133	0.032	0.083	0.055	0.106	**0.733**	0.179	0.083	0.134
srem10	−0.105	−0.033	−0.011	−0.166	−0.031	0.014	−0.173	0.051	**0.589**	0.067	0.051	0.039
srem2	0.059	0.068	−0.024	−0.036	−0.005	0.1	0.019	0.039	**0.709**	0.13	0.091	0.114
srem3	0.004	0.045	−0.092	−0.049	0.083	0.098	0.069	0.098	**0.777**	0.267	0.017	0.175
srem4	0.028	0.018	−0.01	−0.009	0.082	0.108	−0.052	0.086	**0.633**	0.146	0.097	0.104
srem5	−0.013	0.106	−0.032	−0.088	0.092	0.182	−0.024	0.089	**0.785**	0.229	0.085	0.191
srem6	0.053	0.045	−0.108	−0.035	0.044	0.137	−0.026	0.099	**0.716**	0.221	0.084	0.083
srem7	0.047	0.100	−0.055	−0.001	0.074	0.16	−0.031	0.112	**0.76**	0.217	0.074	0.166
srem8	−0.046	0.02	−0.08	−0.096	0.000	0.121	−0.063	0.052	**0.719**	0.196	0.029	0.15
srem9	0.017	0.012	−0.037	−0.056	0.012	0.071	0.057	0.134	**0.71**	0.206	0.111	0.172
sren1	0.088	0.219	0.117	0.047	0.203	0.175	0.185	0.189	0.165	**0.707**	0.328	0.287
sren10	0.004	0.22	0.117	−0.022	0.193	0.221	0.186	0.262	0.163	**0.715**	0.211	0.335
sren2	0.087	0.212	0.07	−0.022	0.179	0.226	0.277	0.21	0.167	**0.694**	0.311	0.351
sren3	0.079	0.200	0.099	0.156	0.22	0.152	0.211	0.319	0.282	**0.74**	0.353	0.306
sren4	0.211	0.289	0.09	0.035	0.183	0.239	0.235	0.271	0.196	**0.698**	0.283	0.33
sren5	0.082	0.26	0.155	0.129	0.269	0.192	0.281	0.201	0.261	**0.757**	0.336	0.286
sren6	0.12	0.244	0.114	0.121	0.193	0.191	0.193	0.292	0.184	**0.709**	0.309	0.303
sren7	0.111	0.331	0.094	0.116	0.254	0.28	0.274	0.283	0.2	**0.757**	0.322	0.341
sren8	0.101	0.213	0.133	0.109	0.204	0.204	0.188	0.253	0.165	**0.74**	0.311	0.344
sren9	0.009	0.22	0.124	0.018	0.207	0.224	0.182	0.278	0.165	**0.705**	0.199	0.37
srow1	0.08	0.181	0.09	0.021	0.091	0.154	0.207	0.312	0.088	0.276	**0.694**	0.245
srow10	0.037	0.137	0.038	−0.03	0.171	0.138	0.084	0.295	0.041	0.135	**0.503**	0.145
srow2	0.041	0.167	0.051	0.008	0.096	0.131	0.232	0.443	0.063	0.366	**0.71**	0.279
srow3	0.088	0.133	0.117	0.115	0.113	0.09	0.174	0.436	0.064	0.299	**0.738**	0.277
srow4	0.121	0.17	0.077	0.005	0.093	0.102	0.161	0.439	0.084	0.295	**0.701**	0.337
srow5	0.007	0.195	0.077	0.008	0.173	0.13	0.174	0.376	0.131	0.308	**0.766**	0.282
srow6	0.18	0.221	0.161	0.073	0.095	0.206	0.237	0.456	0.047	0.322	**0.775**	0.324
srow7	0.1	0.226	0.124	0.055	0.192	0.191	0.215	0.459	0.082	0.313	**0.789**	0.328
srow8	0.17	0.269	0.127	0.122	0.175	0.248	0.252	0.456	0.051	0.333	**0.801**	0.345
srow9	0.037	0.185	0.134	0.044	0.156	0.175	0.16	0.471	0.085	0.283	**0.711**	0.278
srsp1	0.026	0.05	−0.007	−0.047	0.014	0.015	0.118	0.238	0.127	0.277	0.32	**0.737**
srsp10	0.003	0.082	−0.005	−0.083	0.1	0.082	0.05	0.253	0.069	0.223	0.193	**0.568**
srsp2	−0.007	−0.001	0.041	−0.019	−0.026	0.009	0.147	0.242	0.143	0.287	0.274	**0.74**
srsp3	−0.066	0.066	0.025	0	0.104	0.04	0.041	0.317	0.205	0.363	0.291	**0.76**
srsp4	0.058	0.08	0.066	−0.013	0.023	0.096	0.173	0.261	0.15	0.324	0.252	**0.70**
srsp5	−0.025	0.09	−0.014	0.033	0.137	0.038	0.17	0.351	0.203	0.358	0.397	**0.801**
srsp6	0.058	0.159	0.082	0.026	0.053	0.153	0.14	0.286	0.033	0.356	0.274	**0.761**
srsp7	−0.058	0.131	0.007	−0.004	0.109	0.063	0.213	0.306	0.153	0.369	0.332	**0.774**
srsp8	0.053	0.178	0.071	0.02	0.06	0.181	0.17	0.305	0.104	0.365	0.302	**0.765**
srsp9	−0.087	0.038	−0.049	−0.041	0.109	0.016	0.093	0.33	0.222	0.344	0.257	**0.698**

Notes

1 = agreeableness, 2 = bricolage, 3 = conscientiousness, 4 = extroversion, 5 = neuroticism, 6 = openness, 7 = SSR community, 8 = SSR customers, 9 = SSR employees, 10 = SSR environment, 11 = owners, 12 = suppliers.

**TABLE 4:** Construct validity and reliability

Constructs	Cronbach's alpha	Composite reliability	Average variance extracted (AVE)	Discriminant validity
Extroversion	0.829	0.886	0.660	0.812
Agreeableness	0.825	0.884	0.656	0.810
Openness	0.778	0.850	0.531	0.729
Conscientious	0.882	0.914	0.680	0.825
Neuroticism	0.897	0.924	0.710	0.843
Bricolage	0.864	0.894	0.514	0.717
SSR Community	0.888	0.908	0.500	0.706
SSR Customers	0.917	0.929	0.525	0.725
SSR Employees	0.894	0.912	0.512	0.716
SSR Environment	0.898	0.916	0.522	0.722
SSR Owner	0.897	0.916	0.523	0.723
SSR Suppliers	0.903	0.920	0.537	0.733

#### 5.4.1 Convergent validity

It demonstrates the variance explained by items in a particular variable. Our results met the condition (above 0.50) and confirmed that the items explained sufficient variance in their respective construct (Hair et al., 2017).

#### 5.4.2 Discriminant validity

It illustrates if the items explain unique variance or overlapping each other while loaded on a specified variable. Our results displayed desirable values (above 0.70) for all the constructs as recommended by Bagozzi and Yi (1988). The value is higher than the correlation values between the variables, confirming desirable validity (Hulland, 1999).

#### 5.4.3 Composite reliability

It displays the internal consistency and reliability among the items loaded on a specific variable. The threshold of the composite reliability was 0.70 or above, as recommended by Bacon et al. (1995); our research fulfilled this condition.

#### 5.4.4 Cronbach's alpha

This is another test to confirm the internal consistency between the items toward a specific variable. A value greater than or equal to 0.70 delineates satisfactory reliability (Peterson, 1994). Our research met this condition.

### 5.5 Correlations

The relationships between the variables are shown in Table 5. It is seen that extroversion is significantly and positively related to SSR (*r* = 0.020). Agreeableness is also positively related to SSR (*r* = 0.142). Openness displays a positive association (*r* = 0.280), and there is a positive link between conscientiousness and SSR (*r* = 0.121). Neuroticism is also positively associated with SSR (*r* = 0.248). All the personality traits are positively associated with bricolage, and bricolage has a positive association with SSR.

**TABLE 5:** Correlation coefficients

Variables	1	2	3	4	5	6	7	8	9	10	11	12	13	14	15	16
1. Age	1.00															
2. Size	0.23	1.00														
3. Education	0.07	0.17	1.00													
4. Agreeable	0.02	0.05	0.02	1.00												
5. Bricolage	−0.01	0.07	−0.06	0.43	1.00											
6. Conscientious	0.00	−0.01	−0.06	0.23	0.59	1.00										
7. Extroversion	0.08	0.14	0.15	0.37	0.25	0.26	1.00									
8. Neuroticism	−0.01	0.05	0.19	0.28	0.57	0.30	−0.01	1.00								
9. Openness	−0.05	0.02	0.19	0.39	0.80	0.43	−0.02	0.54	1.00							
10. SSR	0.01	0.02	0.09	0.14	0.32	0.12	0.02	0.25	0.28	1.00						
11. SSR Community	0.05	−0.01	0.15	0.20	0.27	0.12	−0.02	0.16	0.21	0.45	1.00					
12. SSR Customers	0.11	0.02	0.09	0.10	0.15	0.04	0.01	0.13	0.14	0.75	0.15	1.00				
13. SSR Employees	−0.10	−0.08	−0.09	0.01	0.06	−0.08	0.02	0.06	0.16	0.32	−0.01	0.13	1.00			
14. SSR Environment	−0.03	−0.01	0.10	0.13	0.33	0.15	0.06	0.29	0.29	0.74	0.31	0.36	0.27	1.00		
15. SSR Owner	0.00	0.04	0.06	0.12	0.26	0.14	−0.01	0.19	0.22	0.78	0.27	0.58	0.10	0.41	1.00	
16. SSR Suppliers	−0.05	−0.04	0.10	−0.01	0.12	0.03	0.01	0.10	0.09	0.70	0.18	0.40	0.20	0.45	0.40	1.00

The *R* ^2^ describes 75% variation in bricolage, and 12% variance in SSR comes from the big five personality traits. The *R* ^2^ is shown in Table 6.

**TABLE 6:** *R* ^2^ and adjusted *R* ^2^

Constructs	*R* ^2^	*R* ^2^ adjusted
Bricolage	0.751	0.746
SSR	0.116	0.083
SSR community	0.198	0.194
SSR customers	0.561	0.559
SSR employees	0.102	0.099
SSR environment	0.552	0.550
SSR owner	0.601	0.599
SSR suppliers	0.495	0.493

### 5.6 Structural model

**FIGURE 3:**
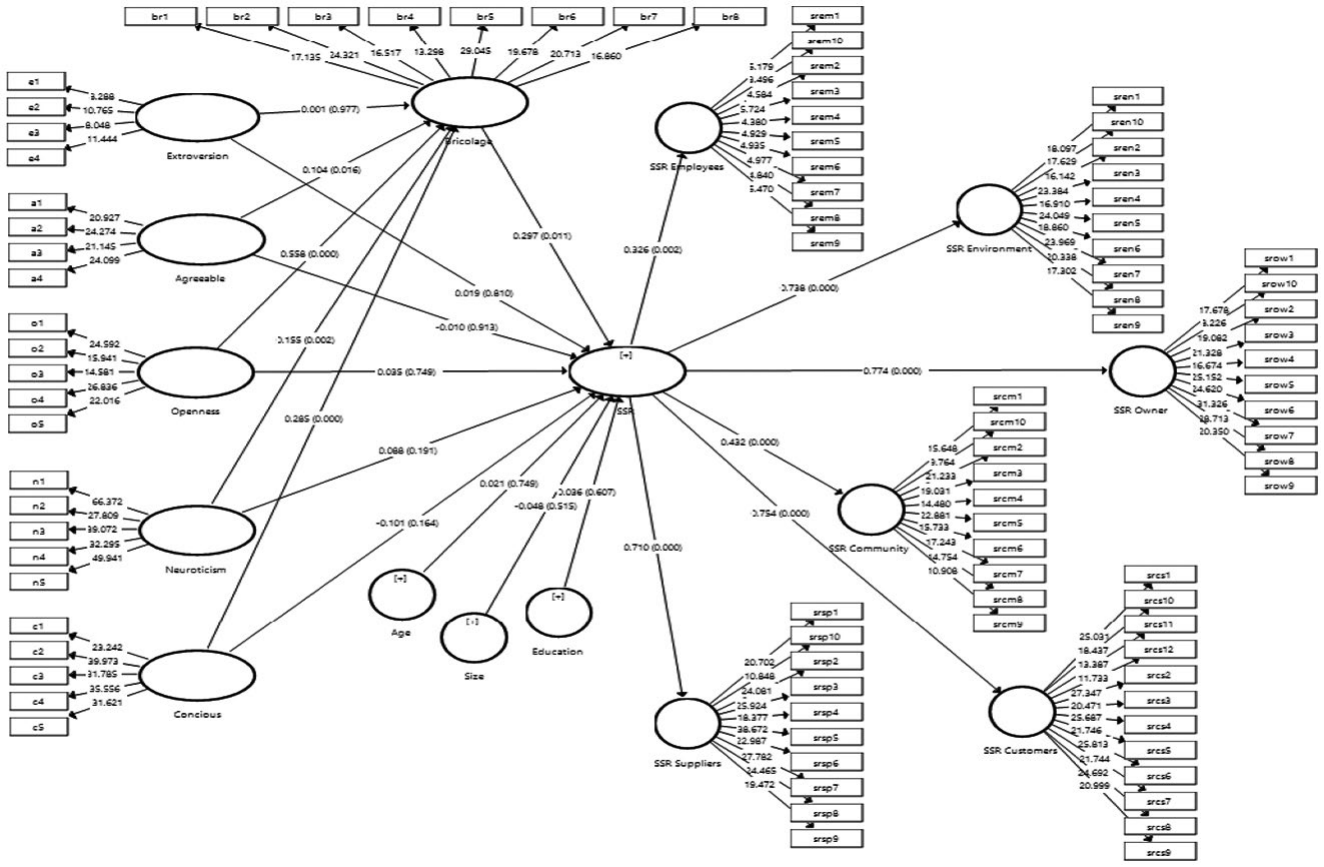
Structural model

The structural model (see Figure 3) in SmartPLS with a resampling approach (bootstrapping 500; 95% confidence interval) was applied to test the hypothesized relationship. We indicated the following second‐order constructs for SSR: SSR toward employees, SSR toward the environment, SSR toward owners, SSR toward the community, SSR toward suppliers, and SSR toward customers. We confirmed the model fitness before explaining the results.

Our results (see Table 7) display that the big five personality traits do not have a significant direct influence on SSR: extroversion (*β* = 0.019, *p* > 0.05), agreeableness (*β* = −0.010, *p* > 0.05), openness (*β* = 0.035, *p* > 0.05), conscientiousness (*β* = −0.101, *p* > 0.05), and neuroticism (*β* = 0.088, *p* > 0.05). They do not support H1–H5.

**TABLE 7:** Hypotheses testing

Paths	Original sample	*T* statistics	*P* values
*Direct effects*			
Age → SSR	0.021	0.320	0.749
Size → SSR	−0.048	0.652	0.515
Education → SSR	0.036	0.515	0.607
Extroversion → SSR	0.019	0.241	0.810
Agreeable → SSR	−0.010	0.109	0.913
Openness → SSR	0.035	0.320	0.749
Conscientious → SSR	−0.101	1.393	0.164
Neuroticism → SSR	0.088	1.310	0.191
Extroversion → Bricolage	0.001	0.028	0.977
Agreeable → Bricolage	0.104	2.422	0.016
Openness → Bricolage	0.558	8.325	0.000
Conscientious → Bricolage	0.285	5.569	0.000
Neuroticism → Bricolage	0.155	3.077	0.002
Bricolage → SSR	0.297	2.563	0.011
SSR → SSR Community	0.432	4.441	0.000
SSR → SSR Customers	0.754	16.883	0.000
SSR → SSR Employees	0.326	3.190	0.002
SSR → SSR Environment	0.738	13.753	0.000
SSR → SSR Owner	0.774	20.141	0.000
SSR → SSR Suppliers	0.710	15.617	0.000
*Indirect effects*			
Extroversion → Bricolage → SSR	0.000	0.027	0.979
Agreeable → Bricolage → SSR	0.031	1.526	0.128
Openness → Bricolage → SSR	0.166	2.370	0.018
Conscientious → Bricolage → SSR	0.085	2.543	0.011
Neuroticism → Bricolage → SSR	0.046	2.082	0.038
*Total effects*			
Age → SSR	0.021	0.320	0.749
Size → SSR	−0.048	0.652	0.515
Education → SSR	0.036	0.515	0.607
Extroversion → SSR	0.020	0.242	0.809
Agreeable → SSR	0.021	0.221	0.825
Openness → SSR	0.201	2.180	0.030
Conscientious → SSR	−0.017	0.233	0.816
Neuroticism → SSR	0.134	2.027	0.043
Bricolage → SSR	0.297	2.563	0.011

All the big five personality traits except extroversion significantly influence bricolage: extroversion (*β* = 0.001, *p* > 0.05), agreeableness (*β* = 0.104, *p* < 0.05), openness (*β* = 0.558, *p* < 0.05), conscientiousness (*β* = 0.285, *p* < 0.05), and neuroticism (*β* = 0.155, *p* < 0.05) which rejected H6 but accepted H7–H10, respectively. Our results reveal that bricolage has a significant influence on SSR (*β* = 0.297, *p* < 0.05), and support H11.

Our results furthermore indicate that the indirect influence of extroversion and agreeable personalities is insignificant on SSR (*β* = 0.000, *p* > 0.05 & *β* = 0.031, *p* < 0.05). This as a result does not support H12 and H13, respectively. It reveals that bricolage does not mediate the paths between extroversion and agreeable personalities and SSR. However, our results also show that the indirect influence of openness, conscientiousness, and neuroticism is significant (*β* = 0.166, *p* < 0.05, *β* = 0.085, *p* < 0.05 & *β* = 0.046, *p* < 0.05). The direct impact became insignificant, supporting H13—H15.

## 6 DISCUSSION AND CONCLUSION

This study examined the influence of the big five personality traits of family SME managers/owners on SSR with a mediating bricolage role. Utilizing data of 245 family‐owned SMEs, we found that SSR activities on average increased during the COVID‐19 shutdown. The distinct findings among family‐owned SMEs make this clear and are indicative of Pakistan.

These findings play a vital role in helping identify what can support stability in Pakistani society for the business industry, for example, especially SMEs. Previous studies have shed light on the psychological factors and personality traits involved in organizational performance, focusing on financial outcomes (Nadkarni & Herrmann, 2010; O’Reilly et al., 2014; Shalender & Yadav, 2019) and CSR activities (Basil & Weber, 2006; Moscato & Hopp, 2019). No study however has reported on the role of the big five personality traits of top management managers in promoting SSR. Moreover, before our study, it was unknown whether any of these traits directly or indirectly influence SSR through bricolage. The present study advances our knowledge of these issues, revealing that some of the traits play a vital role in bricolage, which in turn influences SSR. Taking into account, the contribution of this research to stakeholder theory (Freeman, 2010), we found that top managers and owners actively strive to build favorable relationships with all stakeholders in order to protect the environment. Our research revealed that managers with specific traits can manage resources efficiently (e.g., bricolage), persistently engaging in building a positive relationship with stakeholders to protect the environment and benefit the community. This helps clarify the ambiguity in the literature surrounding this topic and helps understand what kinds of traits are more important when it comes to SSR and helping stakeholders. Studies have yet to consider the psychological traits of managers in stakeholder theory, and have for the most part entirely ignored the bricolage concept.

Our research reveals that the big five personality traits of top managers do not directly influence SSR; this runs counter to our initial assumptions. Our findings are different than several previous studies where a significant relationship between psychological factors and personal motives and the social initiative was reported (e.g., Basil & Weber, 2006; Tran & Von Korflesch, 2016). For instance, Bernardino et al. (2018) examined how individuals with openness to experience, agreeableness, conscientiousness, and that have extroverted personalities are more likely to start new social enterprises. Also our results are different than Hirsh (2010), Hirsh (2014), Pavalache‐Ilie and Cazan (2018) who found significant and direct associations between personality traits pro‐environmental behaviors Our findings are related to Brick and Lewis (2016) who scrutinized that green personality traits indirectly influence environmental friendly behaviors through attitudes toward the natural environment.

Our results furthermore revealed that bricolage mediates the relationship between conscientiousness, neuroticism, and openness and SSR, while bricolage does not play any mediating role between extroversion and agreeable traits and SSR. We found that the indirect paths or direct paths between extroversion, agreeableness, and bricolage and SSR are insignificant, not supporting our suppositions as a result. For instance, our research discloses that managers/owners with extroverted personalities do not bricolage resources. Extroverted people are socially oriented and like to participate in social activities (Amichai‐Hamburger & Vinitzky, 2010; Gosling et al., 2011). Our findings are different from those of Shaw et al. (2016) who claim that extroverted individuals properly allocate resources to perform a given task at a particular time. However, Nguyen et al. (2013) claim that extroverts are more efficient in acquiring resources, while introverts can utilize their mental resources effectively. Similarly, Pickett et al. (2019) also revealed that extroverts deliver a lower level of goal‐oriented performance in an organization. Our results match a meta‐analysis claiming that extroversion is not related to goal‐oriented performance (McCabe et al., 2013). Our results also do not support Ryan and Xenos (2011), Amichai‐Hamburger and Vinitzky (2010), and Gosling et al. (2011) who described how extroverted individuals exhibit high social activity and are engaged in building a wide social network. Additionally, in our study, the surprising results in terms of extroversion's relation to SSR might come from the current COVID‐19 situation that has dismantled groups, teams, and meetings, compelling people to work alone or within a limited circle. Despite hoping to have extroverts around them, they are unable to practice and participate in social activities and programs, strongly contrasting to their usual activities and interests (e.g., they are more outgoing, convivial, and chatty, favoring work with others in a team rather than working alone (Kristof‐Brown et al., 2005)).

We found that agreeable traits are significantly related to bricolage, with our results indicating that managers with agreeable personalities effectively manage their resources to gain maximum benefits, suggesting that agreeable managers do not accept criticism, work in a way to avoid it, and make the people around them happy (Cable & Judge, 2003). We also found that conscientious managers significantly bricolage resources to protect their stakeholders. This is in line with Organ and Ryan (1995) who claimed that conscientious people perform work and achieve tasks while requiring only minimal inputs. Our analysis also revealed that neurotic managers tend to bricolage their resources to provide maximum advantages to society. Our findings do not coincide with Farrukh et al. (2017) who claimed that neurotic employees have a low level of commitment ability.

To summarize, our results illustrate that managers of family‐owned SMEs with conscientious, neurotic, and open personalities effectively utilize existing resources to practice SSR and protect their stakeholders. In other words, managers with high scores of conscientiousness, neuroticism, and openness indirectly contribute to SSR through bricolage.

### 6.1 Managerial implications

Given the increasing COVID‐19 concerns worldwide, our study provides expedient implications for policymakers, top managers, and practitioners. First, by clarifying one of the driving aspects of personality traits, our research examines how the big five personality traits do not directly influence SSR activities during crises. It is seen that enterprises should not only emphasize all the personality traits of top managers but also need managers with personalities who can manage resources (bricolage). In particular, conscientious, neurotic, and open personalities should be concentrated on during crises such as COVID‐19 because it is these types of personalities that effectively utilize existing resources to protect stakeholders. Second, COVID‐19 has impacted all business and non‐business activities, in some cases completely stopping commercial transactions. It has been challenging for enterprises to keep their stakeholders happy with existing resources. From this perspective, our research is an attempt to facilitate enterprises’ response to this challenge and manage existing resources with the help of top managers’ abilities. Third, particularly in Pakistan, COVID‐19 has created a widespread desire for socially driven activity in the business world, with pressure from customers and society expecting enterprises to conduct themselves socially. Irrespective of their size and business, enterprises have shown a great interest in philanthropic action. Our research recommends they bricolage their existing resources. This is possible with managers displaying high levels of conscientiousness, neuroticism, and openness. And managers with extroverted and agreeable personalities can be assigned other tasks even when they are not made responsible for the optimization of existing resources. One idea would be to give them the task of acquiring new external resources. Nguyen et al. (2013) for instance claimed that extroverted managers perform better in the acquisition of external resources because of their social, relationship‐focused behavior.

Policymakers should also give extensive consideration to efficiently utilizing their existing tangible and intangible resources to achieve their SDGs. This will require hiring managers and staff with the ability to bricolage resources in a way providing maximum benefit. Our research sends a clear signal to listed/large firms, showing them their level of involvement in CSR activities compared to SMEs (Udayasankar, 2008). Here they have an opportunity to receive feedback from society and the environment via the right bricolage strategy. Additionally, foreign enterprises can also benefit from these implications to boost their social activities during the COVID‐19 pandemic.

### 6.2 Limitations and future research

This research of course contains caveats that future researchers will have to examine and acknowledge. For instance, the generalizability of the results, although applicable in Pakistan and other neighboring countries, may not be universal. Therefore, family businesses from other (European and Asian) countries should be surveyed to articulate the results in a better/different way. Social desirability biases may also exist in a cross‐sectional data set, thus lacking result validity. To avoid this, we suggest longitudinal data or in‐depth interviews with owners and managers of family firms. Additionally, obtaining responses from other managers (instead of a single respondent) will minimize social desirability bias and its adverse influence on results. The limitations of this research are furthermore in line with the conceptual framework assessing the influence of personality traits on SSR with a mediating role of bricolage. Though standardized traits (e.g., the big five) are considered in this study, the literature has recognized other managerial traits such as overconfidence, emotions, lack of control, and proactive personalities (LeBoutillier & Barry, 2018; Schredl, 2007; Van Aarde et al., 2017). Moreover, in this study, SSR is considered as an outcome variable; this may not be a primary objective of family‐based SMEs. So additional outcomes such as profitability and sustainable competitiveness can and should be considered in future research.

### 6.3 Conclusion

This study examined the influence of the big five personality traits of managers/owners of family‐owned SMEs on SSR with a mediating bricolage role. We used the empirical data of 245 family‐owned SMEs to test the hypothesized model. After applying SmartPLS, we found that the big five personality traits do not directly influence SSR. Nevertheless, all the personality traits except extroversion have a significant influence on bricolage. Additionally, we found that open, conscientious, and neurotic personalities indirectly contribute to SSR through bricolage as a mediator. Our research recommends that SMEs focus on those personalities having high bricolage capability to protect stakeholders, particularly during crises such as the COVID‐19 pandemic. Moreover, family firms need to assign resource management utilization roles to those family members who have open, conscientious, and neurotic personalities. It is after all people with these attributes who can effectively manage existing resources to the protection of stakeholders.

## References

[basr12222-bib-0001] Albats, E. , Alexander, A. , Mahdad, M. , Miller, K. , & Post, G. (2019). Stakeholder management in SME open innovation: Interdependences and strategic actions. Journal of Business Research, 119, 291–301. 10.1016/j.jbusres.2019.07.038

[basr12222-bib-0002] Ali, I. (2019). Personality traits, individual innovativeness and satisfaction with life. Journal of Innovation & Knowledge, 4(1), 38–46.

[basr12222-bib-0003] Amichai‐Hamburger, Y. , & Vinitzky, G. (2010). Social network use and personality. Computers in Human Behavior, 26(6), 1289–1295.

[basr12222-bib-0004] An, W. , Zhang, J. , You, C. , & Guo, Z. (2018). Entrepreneur’s creativity and firm‐level innovation performance: Bricolage as a mediator. Technology Analysis & Strategic Management, 30(7), 838–851.

[basr12222-bib-0005] Anwar, M. (2018). Business model innovation and SMEs performance—Does competitive advantage mediate? International Journal of Innovation Management, 22(7), 1850057.

[basr12222-bib-0006] Anwar, M. , Shah, S. Z. A. , & Khan, S. Z. (2018). The role of personality in SMEs internationalization: Empirical evidence. Review of International Business and Strategy, 28(2), 258–282.

[basr12222-bib-0007] Arbuthnot, J. (1977). The roles of attitudinal and personality variables in the prediction of environmental behavior and knowledge. Environment and Behavior, 9(2), 217–232.

[basr12222-bib-0008] Arregle, J. L. , Hitt, M. A. , Sirmon, D. G. , & Very, P. (2007). The development of organizational social capital: Attributes of family firms. Journal of Management Studies, 44(1), 73–95.

[basr12222-bib-0009] Bacon, D. R. , Sauer, P. L. , & Young, M. (1995). Composite reliability in structural equations modeling. Educational and Psychological Measurement, 55(3), 394–406.

[basr12222-bib-0222] Bagozzi, R. P. , & Yi, Y. (1988). On the evaluation of structural equation models. Journal of the Academy of Marketing Science, 16(1), 74–94.

[basr12222-bib-0204] Baker, T. (2007). Resources in play: Bricolage in the Toy Store (y). Journal of Business Venturing, 22(5), 694–711.

[basr12222-bib-0010] Baker, T. , & Nelson, R. E. (2005). Creating something from nothing: Resource construction through entrepreneurial bricolage. Administrative Science Quarterly, 50(3), 329–366.

[basr12222-bib-0011] Barrena‐Martinez, J. , López‐Fernández, M. , & Romero‐Fernández, P. M. (2019). The link between socially responsible human resource management and intellectual capital. Corporate Social Responsibility and Environmental Management, 26(1), 71–81.

[basr12222-bib-0012] Barrick, M. R. , & Mount, M. K. (1991). The big five personality dimensions and job performance: A meta‐analysis. Personnel Psychology, 44(1), 1–26.

[basr12222-bib-0013] Basil, D. Z. , & Weber, D. (2006). Values motivation and concern for appearances: The effect of personality traits on responses to corporate social responsibility. International Journal of Nonprofit and Voluntary Sector Marketing, 11(1), 61–72.

[basr12222-bib-0014] Beck, T. , Demirguc‐Kunt, A. , & Levine, R. (2005). SMEs, growth, and poverty: Cross‐country evidence. Journal of Economic Growth, 10(3), 199–229.

[basr12222-bib-0015] Berings, D. , & Adriaenssens, S. (2012). The role of business ethics, personality, work values and gender in vocational interests from adolescents. Journal of Business Ethics, 106(3), 325–335.

[basr12222-bib-0016] Bernardino, S. , Santos, J. F. , & Ribeiro, J. C. (2018). Social entrepreneur and gender: What’s personality got to do with it? International Journal of Gender and Entrepreneurship, 10(1), 61–82.

[basr12222-bib-0017] Berry‐Stölzle, T. R. , Eastman, E. M. , & Xu, J. (2018). CEO overconfidence and earnings management: Evidence from property‐liability insurers’ loss reserves. North American Actuarial Journal, 22(3), 380–404.

[basr12222-bib-0018] Bocquet, R. , Le Bas, C. , Mothe, C. , & Poussing, N. (2019). Strategic CSR for innovation in SMEs: Does diversity matter? Long Range Planning, 52(6), 101913.

[basr12222-bib-0210] Borden, R. J. , & Francis, J. L. (1978). Who cares about ecology? Personality and sex differences in environmental concern 1. Journal of Personality, 46(1), 190–203. 650379 10.1111/j.1467-6494.1978.tb00610.x

[basr12222-bib-0207] Bowen, H. R. (1953). Social responsibility of the businessman. Harper and Row.

[basr12222-bib-0019] Bretas, V. P. G. , & Alon, I. (2020). The impact of COVID‐19 on franchising in emerging markets: An example from Brazil. Global Business and Organizational Excellence, 39(6), 6–16.

[basr12222-bib-0020] Brick, C. , & Lewis, G. J. (2016). Unearthing the “green” personality: Core traits predict environmentally friendly behavior. Environment and Behavior, 48(5), 635–658.

[basr12222-bib-0021] Brown, S. , & Taylor, K. (2014). Household finances and the ‘Big Five’ personality traits. Journal of Economic Psychology, 45, 197–212.

[basr12222-bib-0022] Browne, M. W. , & Cudeck, R. (1992). Alternative ways of assessing model fit. Sociological Methods & Research, 21(2), 230–258.

[basr12222-bib-0023] Busic‐Sontic, A. , Czap, N. V. , & Fuerst, F. (2017). The role of personality traits in green decision‐making. Journal of Economic Psychology, 62, 313–328.

[basr12222-bib-0024] Cable, D. M. , & Judge, T. A. (2003). Managers’ upward influence tactic strategies: The role of manager personality and supervisor leadership style. Journal of Organizational Behavior: The International Journal of Industrial, Occupational and Organizational Psychology and Behavior, 24(2), 197–214.

[basr12222-bib-0025] Carmeli, A. , Dothan, A. , & Boojihawon, D. K. (2020). Resilience of sustainability‐oriented and financially‐driven organizations. Business Strategy and the Environment, 29(1), 154–169.

[basr12222-bib-0026] Carpenter, M. A. , Geletkanycz, M. A. , & Sanders, W. G. (2004). Upper echelons research revisited: Antecedents, elements, and consequences of top management team composition. Journal of Management, 30(6), 749–778.

[basr12222-bib-0027] Carroll, A. B. , Lipartito, K. J. , Post, J. E. , & Werhane, P. H. (2012). Corporate responsibility: The American experience. Cambridge University Press.

[basr12222-bib-0028] Cheong, C. W. , Sinnakkannu, J. , & Ramasamy, S. (2017). Reactive or proactive? Investor sentiment as a driver of corporate social responsibility. Research in International Business and Finance, 42, 572–582.

[basr12222-bib-0029] Choi, J. S. , Kwak, Y. M. , & Choe, C. (2010). Corporate social responsibility and corporate financial performance: Evidence from Korea. Australian Journal of Management, 35(3), 291–311.

[basr12222-bib-0030] Costa, P. T. Jr , & McCrae, R. R. (1992). Four ways five factors are basic. Personality and Individual Differences, 13(6), 653–665.

[basr12222-bib-0217] Desa, G. (2012). Resource mobilization in international social entrepreneurship: Bricolage as a mechanism of institutional transformation. Entrepreneurship Theory and Practice, 36(4), 727–751.

[basr12222-bib-0206] Duymedjian, R. , & Rüling, C. C. (2010). Towards a foundation of bricolage in organization and management theory. Organization Studies, 31(2), 133–151.

[basr12222-bib-0031] Eggers, F. (2020). Masters of disasters? Challenges and opportunities for SMEs in times of crisis. Journal of Business Research, 116, 199–208. 32501306 10.1016/j.jbusres.2020.05.025 PMC7247971

[basr12222-bib-0032] El Baz, J. , Laguir, I. , Marais, M. , & Staglianò, R. (2016). Influence of national institutions on the corporate social responsibility practices of small‐and medium‐sized enterprises in the food‐processing industry: Differences between France and Morocco. Journal of Business Ethics, 134(1), 117–133.

[basr12222-bib-0033] Endrikat, J. , De Villiers, C. , Guenther, T. W. , & Guenther, E. M. (2020). Board characteristics and corporate social responsibility: A meta‐analytic investigation. Business & Society, 000765032093063. 10.1177/0007650320930638

[basr12222-bib-0034] Esmaeelinezhad, O. , & Afrazeh, A. (2018). Linking personality traits and individuals’ knowledge management behavior. Aslib Journal of Information Management, 70(3), 234–251.

[basr12222-bib-0211] Fang, R. , Landis, B. , Zhang, Z. , Anderson, M. H. , Shaw, J. D. , & Kilduff, M. (2015). Integrating personality and social networks: A meta‐analysis of personality, network position, and work outcomes in organizations. Organization Science, 26(4), 1243–1260.

[basr12222-bib-0035] Farrukh, M. , Ying, C. W. , & Mansori, S. (2017). Organizational commitment: An empirical analysis of personality traits. Journal of Work‐Applied Management, 9(1), 18–34.

[basr12222-bib-0221] Ferneley, E. , & Bell, F. (2006). Using bricolage to integrate business and information technology innovation in SMEs. Technovation, 26(2), 232–241.

[basr12222-bib-0036] Fraj, E. , & Martinez, E. (2006). Influence of personality on ecological consumer behaviour. Journal of Consumer Behaviour: An International Research Review, 5(3), 167–181.

[basr12222-bib-0037] Freeman, R. E. (2010). Strategic management: A stakeholder approach. Cambridge University Press.

[basr12222-bib-0038] Freeman, R. E. , & McVea, J. (2001). A stakeholder approach to strategic management. The Blackwell Handbook of Strategic Management, 189–207.

[basr12222-bib-0039] García‐Sánchez, I. M. , Aibar‐Guzmán, B. , Aibar‐Guzmán, C. , & Azevedo, T. C. (2020). CEO ability and sustainability disclosures: The mediating effect of corporate social responsibility performance. Corporate Social Responsibility and Environmental Management, 27(4), 1565–1577. 10.1002/csr.1905

[basr12222-bib-0040] Garriga, E. , & Melé, D. (2004). Corporate social responsibility theories: Mapping the territory. Journal of Business Ethics, 53(1–2), 51–71.

[basr12222-bib-0041] George, D. (2011). SPSS for windows step by step: A simple study guide and reference, 17.0 update, 10/e. Pearson Education India.

[basr12222-bib-0042] Goldberg, L. R. (1993). The structure of personality traits: Vertical and horizontal aspects. In D. C. Funder , R. D. Parke , C. Tomlinson-Keasey , & K. Widaman (Eds.), Studying lives through time: Personality and development. APA science Vols. (pp. 169–188). American Psychological Association.

[basr12222-bib-0043] Gosling, S. D. , Augustine, A. A. , Vazire, S. , Holtzman, N. , & Gaddis, S. (2011). Manifestations of personality in online social networks: Self‐reported Facebook‐related behaviors and observable profile information. Cyberpsychology, Behavior, and Social Networking, 14(9), 483–488. 21254929 10.1089/cyber.2010.0087 PMC3180765

[basr12222-bib-0044] Graafland, J. , & Noorderhaven, N. (2020). Technological competition, innovation motive and corporate social responsibility: Evidence from top managers of European SMEs. De Economist, 168(1), 1–22.

[basr12222-bib-0045] Granados, M. L. , Mohamed, S. , & Hlupic, V. (2017). Knowledge management activities in social enterprises: Lessons for small and non‐profit firms. Journal of Knowledge Management, 21(2), 376–396.

[basr12222-bib-0046] Grimstad, S. M. F. , Glavee‐Geo, R. , & Fjørtoft, B. E. (2020). SMEs motivations for CSR: An exploratory study. European Business Review, 32(4), 553–572.

[basr12222-bib-0047] Gupta, A. , Nadkarni, S. , & Mariam, M. (2019). Dispositional sources of managerial discretion: CEO ideology, CEO personality, and firm strategies. Administrative Science Quarterly, 64(4), 855–893.

[basr12222-bib-0048] Hair, J. F. Jr , Matthews, L. M. , Matthews, R. L. , & Sarstedt, M. (2017). PLS‐SEM or CB‐SEM: Updated guidelines on which method to use. International Journal of Multivariate Data Analysis, 1(2), 107–123.

[basr12222-bib-0209] Hambrick, D. C. (2007). Upper echelons theory: An update. Academy of Management Review, 32(2), 334–343.

[basr12222-bib-0049] Hirsh, J. B. (2010). Personality and environmental concern. Journal of Environmental Psychology, 30(2), 245–248.

[basr12222-bib-0050] Hirsh, J. B. (2014). Environmental sustainability and national personality. Journal of Environmental Psychology, 38, 233–240.

[basr12222-bib-0051] Hirsh, J. B. , & Dolderman, D. (2007). Personality predictors of consumerism and environmentalism: A preliminary study. Personality and Individual Differences, 43(6), 1583–1593.

[basr12222-bib-0052] Hooi, H. C. , Ahmad, N. H. , Amran, A. , & Rahman, S. A. (2016). The functional role of entrepreneurial orientation and entrepreneurial bricolage in ensuring sustainable entrepreneurship. Management Research Review, 39(12), 1616–1638.

[basr12222-bib-0053] Hossain, M. U. , & Asheq, A. A. (2020). Do leadership orientation and proactive personality influence social entrepreneurial intention? International Journal of Management and Enterprise Development, 19(2), 109–125.

[basr12222-bib-0054] Howard, P. J. , & Howard, J. M. (1995). The Big Five Quickstart: An introduction to the five‐factor model of personality for human resource professionals. https://eric.ed.gov/?id=ED384754

[basr12222-bib-0055] Hughes, D. J. , Rowe, M. , Batey, M. , & Lee, A. (2012). A tale of two sites: Twitter vs. Facebook and the personality predictors of social media usage. Computers in Human Behavior, 28(2), 561–569.

[basr12222-bib-0223] Hulland, J. (1999). Use of partial least squares (PLS) in strategic management research: A review of four recent studies. Strategic Management Journal, 20(2), 195–204.

[basr12222-bib-0056] Ilyas, S. , Hu, Z. , & Wiwattanakornwong, K. (2020). Unleashing the role of top management and government support in green supply chain management and sustainable development goals. Environmental Science and Pollution Research, 27(8), 8210–8223. 10.1007/s11356-019-07268-3 31900776

[basr12222-bib-0057] İrengün, O. , & Arıkboğa, Ş. (2015). The effect of personality traits on social entrepreneurship intentions: A field research. Procedia‐Social and Behavioral Sciences, 195, 1186–1195.

[basr12222-bib-0058] Jamali, D. (2008). A stakeholder approach to corporate social responsibility: A fresh perspective into theory and practice. Journal of Business Ethics, 82(1), 213–231.

[basr12222-bib-0059] Javed, M. , Rashid, M. A. , & Hussain, G. (2017). Well‐governed responsibility spurs performance. Journal of Cleaner Production, 166, 1059–1073.

[basr12222-bib-0060] Jia, X. (2020). Corporate social responsibility activities and firm performance: The moderating role of strategic emphasis and industry competition. Corporate Social Responsibility and Environmental Management, 27(1), 65–73.

[basr12222-bib-0061] Jiang, F. , Zalan, T. , Herman, H. M. , & Shen, J. (2018). Mapping the relationship among political ideology, CSR mindset, and CSR strategy: A contingency perspective applied to Chinese managers. Journal of Business Ethics, 147(2), 419–444.

[basr12222-bib-0062] Jones, T. M. , Harrison, J. S. , & Felps, W. (2018). How applying instrumental stakeholder theory can provide sustainable competitive advantage. Academy of Management Review, 43(3), 371–391.

[basr12222-bib-0063] Kannampuzha, M. J. , & Suoranta, M. (2016). Bricolage in the marketing efforts of a social enterprise. Journal of Research in Marketing and Entrepreneurship, 18(2), 176–196.

[basr12222-bib-0064] Khan, S. Z. , Yang, Q. , Khan, N. U. , Kherbachi, S. , & Huemann, M. (2020). Sustainable social responsibility toward multiple stakeholders as a trump card for small and medium‐sized enterprise performance (evidence from China). Corporate Social Responsibility and Environmental Management, 27(1), 95–108.

[basr12222-bib-0065] Khan, S. Z. , Yang, Q. , & Waheed, A. (2019). Investment in intangible resources and capabilities spurs sustainable competitive advantage and firm performance. Corporate Social Responsibility and Environmental Management, 26(2), 285–295.

[basr12222-bib-0066] Khattak, M. S. (2020). Does access to domestic finance and international finance contribute to sustainable development goals? Implications for policymakers. Journal of Public Affairs, 20(2). 10.1002/pa.2024

[basr12222-bib-0067] Klein, S. A. , Heck, D. W. , Reese, G. , & Hilbig, B. E. (2019). On the relationship between Openness to Experience, political orientation, and pro‐environmental behavior. Personality and Individual Differences, 138, 344–348.

[basr12222-bib-0068] Knight, H. , Megicks, P. , Agarwal, S. , & Leenders, M. A. A. M. (2019). Firm resources and the development of environmental sustainability among small and medium‐sized enterprises: Evidence from the Australian wine industry. Business Strategy and the Environment, 28(1), 25–39.

[basr12222-bib-0069] Kristof‐Brown, A. , Barrick, M. R. , & Kay Stevens, C. (2005). When opposites attract: A multi‐sample demonstration of complementary person‐team fit on extraversion. Journal of Personality, 73(4), 935–958. 15958140 10.1111/j.1467-6494.2005.00334.x

[basr12222-bib-0070] Kwong, C. , Tasavori, M. , & Wun‐mei Cheung, C. (2017). Bricolage, collaboration and mission drift in social enterprises. Entrepreneurship & Regional Development, 29(7–8), 609–638.

[basr12222-bib-0071] LeBoutillier, N. , & Barry, R. (2018). Psychological mindedness, personality and creative cognition. Creativity Research Journal, 30(1), 78–84.

[basr12222-bib-0072] Lee, G. , Cho, S. Y. , Arthurs, J. , & Lee, E. K. (2020). Celebrity CEO, identity threat, and impression management: Impact of celebrity status on corporate social responsibility. Journal of Business Research, 111, 69–84.

[basr12222-bib-0203] Lévi‐Strauss, C. (1966). The savage mind. University of Chicago Press.

[basr12222-bib-0073] Liang, C. T. , Peng, L. P. , Yao, S. N. , & Liang, C. (2015). Developing a social enterprise performance scale and examining the relationship between entrepreneurs’ personality traits and their perceived enterprise performance. Journal of Entrepreneurship, Management and Innovation, 11(3), 89–116.

[basr12222-bib-0215] Lim, P. S. , Abdullah, M. N. , & L. Y. , (2012). Relationship between Big‐five Personality Domains and Students' Academic Achievement. Pertanika Journal of Social Sciences & Humanities, 20(4).

[basr12222-bib-0075] Lindgreen, A. , Swaen, V. , Maon, F. , & Pedersen, E. R. (2009). The many and the few: Rounding up the SMEs that manage CSR in the supply chain. Supply Chain Management: An International Journal, 14(2), 109–116.

[basr12222-bib-0076] Linna, P. (2013). Bricolage as a means of innovating in a resource‐scarce environment: A study of innovator‐entrepreneurs at the BOP. Journal of Developmental Entrepreneurship, 18(03), 1350015.

[basr12222-bib-0077] Liu, Y. , Gulzar, M. A. , Zhang, Z. , & Yang, Q. (2018). Do interaction and education moderate top management team age heterogeneity and corporate social responsibility? Social Behavior and Personality: An International Journal, 46(12), 2063–2079.

[basr12222-bib-0078] Lu, Y. , Wu, J. , Peng, J. , & Lu, L. (2020). The perceived impact of the Covid‐19 epidemic: Evidence from a sample of 4807 SMEs in Sichuan Province, China. Environmental Hazards, 19(4), 323–340.

[basr12222-bib-0079] MacKenzie, S. B. , & Podsakoff, P. M. (2012). Common method bias in marketing: Causes, mechanisms, and procedural remedies. Journal of Retailing, 88(4), 542–555.

[basr12222-bib-0212] Malhotra, S. , Reus, T. H. , Zhu, P. , & Roelofsen, E. M. (2018). The acquisitive nature of extraverted CEOs. Administrative Science Quarterly, 63(2), 370–408.

[basr12222-bib-0080] Mandal, D. , McQueen, R. J. , Dillon, S. , & Rastrick, K. (2017). The influence of small enterprise owner personality traits on Facebook use. Small Enterprise Research, 24(2), 120–131.

[basr12222-bib-0081] Mansfield, E. R. , & Helms, B. P. (1982). Detecting multicollinearity. The American Statistician, 36(3a), 158–160.

[basr12222-bib-0082] Markowitz, E. M. , Goldberg, L. R. , Ashton, M. C. , & Lee, K. (2012). Profiling the “pro‐environmental individual”: A personality perspective. Journal of Personality, 80(1), 81–111. 21241310 10.1111/j.1467-6494.2011.00721.x PMC3188340

[basr12222-bib-0083] Martín‐de Castro, G. , Amores‐Salvadó, J. , & Navas‐López, J. E. (2016). Environmental management systems and firm performance: Improving firm environmental policy through stakeholder engagement. Corporate Social Responsibility and Environmental Management, 23(4), 243–256.

[basr12222-bib-0084] Matzler, K. , Renzl, B. , Mooradian, T. , von Krogh, G. , & Mueller, J. (2011). Personality traits, affective commitment, documentation of knowledge, and knowledge sharing. The International Journal of Human Resource Management, 22(02), 296–310.

[basr12222-bib-0085] McCabe, K. O. , Van Yperen, N. W. , Elliot, A. J. , & Verbraak, M. (2013). Big Five personality profiles of context‐specific achievement goals. Journal of Research in Personality, 47(6), 698–707.

[basr12222-bib-0213] McCrae, R. R. , & Costa, P. T. Jr (1997). Personality trait structure as a human universal. American Psychologist, 52(5), 509. 9145021 10.1037//0003-066x.52.5.509

[basr12222-bib-0208] McGuire, J. , Dow, S. , & Argheyd, K. (2003). CEO incentives and corporate social performance. Journal of Business Ethics, 45(4), 341–359.

[basr12222-bib-0086] Memon, A. , Yong An, Z. , & Memon, M. Q. (2020). Does financial availability sustain financial, innovative, and environmental performance? Relation via opportunity recognition. Corporate Social Responsibility and Environmental Management, 27(2), 562–575.

[basr12222-bib-0214] Meyer, R. D. , Dalal, R. S. , José, I. J. , Hermida, R. , Chen, T. R. , Vega, R. P. , Brooks, C. K. , & Khare, V. P. (2014). Measuring job‐related situational strength and assessing its interactive effects with personality on voluntary work behavior. Journal of Management, 40(4), 1010–1041.

[basr12222-bib-0087] Milfont, T. L. , & Sibley, C. G. (2012). The big five personality traits and environmental engagement: Associations at the individual and societal level. Journal of Environmental Psychology, 32(2), 187–195.

[basr12222-bib-0088] Moscato, D. , & Hopp, T. (2019). Natural born cynics? The role of personality characteristics in consumer skepticism of corporate social responsibility behaviors. Corporate Reputation Review, 22(1), 26–37.

[basr12222-bib-0089] Myers, S. D. , Sen, S. , & Alexandrov, A. (2010). The moderating effect of personality traits on attitudes toward advertisements: A contingency framework. Management & Marketing, 5(3), 3.

[basr12222-bib-0090] Myung, J. K. , Choi, Y. H. , & Kim, J. D. (2017). Effects of CEOs’ negative traits on corporate social responsibility. Sustainability, 9(4), 543.

[basr12222-bib-0091] Mzembe, A. N. , Novakovic, Y. , Melissen, F. , & Kamanga, G. (2019). Institutional bricolage as an antecedent of social value creation in a developing country's tourism and hospitality industry. Corporate Social Responsibility and Environmental Management, 26(4), 997–1008.

[basr12222-bib-0092] Nadkarni, S. , & Herrmann, P. O. L. (2010). CEO personality, strategic flexibility, and firm performance: The case of the Indian business process outsourcing industry. Academy of Management Journal, 53(5), 1050–1073.

[basr12222-bib-0093] Nga, J. K. H. , & Shamuganathan, G. (2010). The influence of personality traits and demographic factors on social entrepreneurship start up intentions. Journal of Business Ethics, 95(2), 259–282.

[basr12222-bib-0094] Nguyen, C. , Satterfield, K. , Bellows, B. , McKnight, P. , & Shaw, T. H. (2013). Assessing resource utilization during vigilance using Transcranial Doppler: The effects of extraversion. In Proceedings of the human factors and ergonomics society annual meeting (Vol. 57, No. 1, pp. 818–822). SAGE Publications.

[basr12222-bib-0095] O’Reilly, C. A. III , Caldwell, D. F. , Chatman, J. A. , & Doerr, B. (2014). The promise and problems of organizational culture: CEO personality, culture, and firm performance. Group & Organization Management, 39(6), 595–625.

[basr12222-bib-0096] Obschonka, M. , Schmitt‐Rodermund, E. , Silbereisen, R. K. , Gosling, S. D. , & Potter, J. (2013). The regional distribution and correlates of an entrepreneurship‐prone personality profile in the United States, Germany, and the United Kingdom: A socioecological perspective. Journal of Personality and Social Psychology, 105(1), 104–122. 23586410 10.1037/a0032275

[basr12222-bib-0097] Organ, D. W. (1994). Personality and organizational citizenship behavior. Journal of Management, 20(2), 465–478.

[basr12222-bib-0224] Organ, D. W. , & Ryan, K. (1995). A meta‐analytic review of attitudinal and dispositional predictors of organizational citizenship behavior. Personnel Psychology, 48(4), 775–802.

[basr12222-bib-0098] Parmar, B. L. , Freeman, R. E. , Harrison, J. S. , Wicks, A. C. , Purnell, L. , & De Colle, S. (2010). Stakeholder theory: The state of the art. Academy of Management Annals, 4(1), 403–445.

[basr12222-bib-0099] Paul, K. , Meyskens, M. , & Robbins, S. (2011). Components of a global mindset: Corporate social responsibility and cross‐cultural sensitivity. Journal of International Business and Cultural Studies, 5, 1.

[basr12222-bib-0100] Pavalache‐Ilie, M. , & Cazan, A. M. (2018). Personality correlates of pro‐environmental attitudes. International Journal of Environmental Health Research, 28(1), 71–78. 29360388 10.1080/09603123.2018.1429576

[basr12222-bib-0101] Peng, M. W. , & Luo, Y. (2000). Managerial ties and firm performance in a transition economy: The nature of a micro‐macro link. Academy of Management Journal, 43(3), 486–501.

[basr12222-bib-0102] Pérez, A. , & del Bosque, I. R. (2017). Personal traits and customer responses to CSR perceptions in the banking sector. International Journal of Bank Marketing, 35(1), 128–146.

[basr12222-bib-0103] Peterson, R. A. (1994). A meta‐analysis of Cronbach's coefficient alpha. Journal of Consumer Research, 21(2), 381–391.

[basr12222-bib-0104] Pickett, J. , Hofmans, J. , & De Fruyt, F. (2019). Extraversion and performance approach goal orientation: An integrative approach to personality. Journal of Research in Personality, 82, 103846.

[basr12222-bib-0105] Porter, M. E. , & Kramer, M. R. (2006). The link between competitive advantage and corporate social responsibility. Harvard Business Review, 84(12), 78–92. 17183795

[basr12222-bib-0106] Ratten, V. (2020). Coronavirus and international business: An entrepreneurial ecosystem perspective. Thunderbird International Business Review, 62(5), 629–634.

[basr12222-bib-0201] Robbins, S. P. , & Judge, T. A. (2016). Organizational behavior (global ed.). Pearson Education UK.

[basr12222-bib-0107] Ryan, T. , & Xenos, S. (2011). Who uses Facebook? An investigation into the relationship between the Big Five, shyness, narcissism, loneliness, and Facebook usage. Computers in Human Behavior, 27(5), 1658–1664.

[basr12222-bib-0108] Saha, R. , Cerchione, R. , Singh, R. , & Dahiya, R. (2020). Effect of ethical leadership and corporate social responsibility on firm performance: A systematic review. Corporate Social Responsibility and Environmental Management, 27(2), 409–429.

[basr12222-bib-0109] Sajko, M. , Boone, C. , & Buyl, T. (2020). CEO greed, corporate social responsibility, and organizational resilience to systemic shocks. Journal of Management, 0149206320902528.

[basr12222-bib-0110] Salunke, S. , Weerawardena, J. , & McColl‐Kennedy, J. R. (2013). Competing through service innovation: The role of bricolage and entrepreneurship in project‐oriented firms. Journal of Business Research, 66(8), 1085–1097.

[basr12222-bib-0202] Schmitt, D. B. (Ed.). (2013). Advances in accounting behavioral research. Emerald Group Publishing UK.

[basr12222-bib-0111] Schmitt, D. P. , Allik, J. , McCrae, R. R. , & Benet‐Martínez, V. (2007). The geographic distribution of Big Five personality traits: Patterns and profiles of human self‐description across 56 nations. Journal of cross‐cultural Psychology, 38(2), 173–212.

[basr12222-bib-0112] Schredl, M. (2007). Personality correlates of flying dreams. Imagination, Cognition and Personality, 27(2), 129–137.

[basr12222-bib-0113] Schroeder, M. A. , Lander, J. , & Levine‐Silverman, S. (1990). Diagnosing and dealing with multicollinearity. Western Journal of Nursing Research, 12(2), 175–187. 2321373 10.1177/019394599001200204

[basr12222-bib-0114] Senyard, J. , Baker, T. , Steffens, P. , & Davidsson, P. (2014). Bricolage as a path to innovativeness for resource‐constrained new firms. Journal of Product Innovation Management, 31(2), 211–230.

[basr12222-bib-0115] Shad, M. K. , Lai, F. W. , Fatt, C. L. , Klemeš, J. J. , & Bokhari, A. (2019). Integrating sustainability reporting into enterprise risk management and its relationship with business performance: A conceptual framework. Journal of Cleaner Production, 208, 415–425.

[basr12222-bib-0116] Shalender, K. , & Yadav, R. K. (2019). Strategic flexibility, manager personality, and firm performance: The case of Indian Automobile Industry. Global Journal of Flexible Systems Management, 20(1), 77–90.

[basr12222-bib-0117] Shaw, T. H. , Nguyen, C. , Satterfield, K. , Ramirez, R. , & McKnight, P. E. (2016). Cerebral hemovelocity reveals differential resource allocation strategies for extraverts and introverts during vigilance. Experimental Brain Research, 234(2), 577–585. 26563163 10.1007/s00221-015-4481-8

[basr12222-bib-0118] Singh, S. K. , Chen, J. , Del Giudice, M. , & El‐Kassar, A. N. (2019). Environmental ethics, environmental performance, and competitive advantage: Role of environmental training. Technological Forecasting and Social Change, 146, 203–211.

[basr12222-bib-0119] Sivathanu, B. , & Pillai, R. (2019). An empirical study on entrepreneurial bricolage behavior for sustainable enterprise performance of startups. Journal of Entrepreneurship in Emerging Economies, 12(1), 34–57.

[basr12222-bib-0120] Soliño, M. , & Farizo, B. A. (2014). Personal traits underlying environmental preferences: A discrete choice experiment. PLoS One, 9(2), e89603. 24586905 10.1371/journal.pone.0089603 PMC3930749

[basr12222-bib-0218] Steffens, P. , & Senyard, J. (2009). Linking resource acquisition and development processes to resource‐based advantage: Bricolage and the resource‐based view. In 2009 Babson College Entrepreneurship Research Conference (pp. 1–14).

[basr12222-bib-0121] Su, Z. , Yang, J. , & Wang, Q. (2020). The effects of top management team heterogeneity and shared vision on entrepreneurial bricolage in new ventures: An attention‐based view. IEEE Transactions on Engineering Management, 1–14. 10.1109/TEM.2020.2984628

[basr12222-bib-0122] Tang, J. H. , Chen, M. C. , Yang, C. Y. , Chung, T. Y. , & Lee, Y. A. (2016). Personality traits, interpersonal relationships, online social support, and Facebook addiction. Telematics and Informatics, 33(1), 102–108.

[basr12222-bib-0123] Tran, A. T. , & Von Korflesch, H. (2016). A conceptual model of social entrepreneurial intention based on the social cognitive career theory. Asia Pacific Journal of Innovation and Entrepreneurship, 10(1), 17–38.

[basr12222-bib-0124] Udayasankar, K. (2008). Corporate social responsibility and firm size. Journal of Business Ethics, 83(2), 167–175.

[basr12222-bib-0125] Van Aarde, N. , Meiring, D. , & Wiernik, B. M. (2017). The validity of the Big Five personality traits for job performance: Meta‐analyses of South African studies. International Journal of Selection and Assessment, 25(3), 223–239.

[basr12222-bib-0126] Waller, D. S. , & Lanis, R. (2009). Corporate social responsibility (CSR) disclosure of advertising agencies: An exploratory analysis of six holding companies’ annual reports. Journal of Advertising, 38(1), 109–122.

[basr12222-bib-0127] Wang, J. L. , Jackson, L. A. , Zhang, D. J. , & Su, Z. Q. (2012). The relationships among the Big Five Personality factors, self‐esteem, narcissism, and sensation‐seeking to Chinese University students’ uses of social networking sites (SNSs). Computers in Human Behavior, 28(6), 2313–2319.

[basr12222-bib-0128] Wang, S. , & Chen, X. (2020). Recognizing CEO personality and its impact on business performance: Mining linguistic cues from social media. Information & Management, 57(5), 103173.

[basr12222-bib-0129] Ward, R. , Sreenivas, S. , Read, J. , Saunders, K. E. , & Rogers, R. D. (2017). The role of serotonin in personality inference: Tryptophan depletion impairs the identification of neuroticism in the face. Psychopharmacology (Berl), 234(14), 2139–2147. 28488040 10.1007/s00213-017-4619-4 PMC5486943

[basr12222-bib-0130] Wee, J. , Jang, S. , Lee, J. , & Jang, W. (2017). The influence of depression and personality on social networking. Computers in Human Behavior, 74, 45–52.

[basr12222-bib-0131] Witell, L. , Gebauer, H. , Jaakkola, E. , Hammedi, W. , Patricio, L. , & Perks, H. (2017). A bricolage perspective on service innovation. Journal of Business Research, 79, 290–298.

[basr12222-bib-0132] Withisuphakorn, P. , & Jiraporn, P. (2016). The effect of firm maturity on corporate social responsibility (CSR): Do older firms invest more in CSR? Applied Economics Letters, 23(4), 298–301.

[basr12222-bib-0133] Yadav, P. L. , Han, S. H. , & Kim, H. (2017). Sustaining competitive advantage through corporate environmental performance. Business Strategy and the Environment, 26(3), 345–357.

[basr12222-bib-0134] Yan, S. , Hu, B. , Liu, G. , Ru, X. , & Wu, Q. (2020). Top management team boundary‐spanning behaviour, bricolage, and business model innovation. Technology Analysis & Strategic Management, 32(5), 561–573.

[basr12222-bib-0220] Zain, M. M. , Mohammad, R. , & Alwi, M. R. (2006). Malaysian corporate responsibility disclosure: Miscommunication between providers and users of information? Social Responsibility Journal, 2(1), 96–103.

[basr12222-bib-0135] Zhao, J. , Huang, Y. , Xi, X. , & Wang, S. (2020). How knowledge heterogeneity influences business model design: Mediating effects of strategic learning and bricolage. International Entrepreneurship and Management Journal, 1–31.

[basr12222-bib-0136] Zhu, Y. , Sun, L. Y. , & Leung, A. S. (2014). Corporate social responsibility, firm reputation, and firm performance: The role of ethical leadership. Asia Pacific Journal of Management, 31(4), 925–947.

